# Contrasting response of soil microbiomes to long-term fertilization in various highland cropping systems

**DOI:** 10.1038/s43705-023-00286-w

**Published:** 2023-08-18

**Authors:** Weibo Kong, Liping Qiu, Satoshi Ishii, Xiaoxu Jia, Fuyuan Su, Yu Song, Mingde Hao, Mingan Shao, Xiaorong Wei

**Affiliations:** 1 State Key Laboratory of Soil Erosion and Dryland Farming on the Loess Plateau, Northwest A&F University, Yangling, 712100 Shaanxi China; 2 Research Center of Soil and Water Conservation and Ecological Environment, Ministry of Education, Chinese Academy of Sciences, Yangling, 712100 China; 3 BioTechnology Institute, University of Minnesota, St. Paul, MN 55108 USA; 4 Department of Soil, Water, and Climate, University of Minnesota, St. Paul, MN 55108 USA; 5 Key Laboratory of Ecosystem Network Observation and Modeling, Institute of Geographic Sciences and Natural Resources Research, Chinese Academy of Sciences, 100101 Beijing, China; 6 College of Natural Resources and Environment, Northwest A&F University, Yangling, 712100 China; 7 CAS Center for Excellence in Quaternary Science and Global Change, Xi’an, 710061 Shaanxi China

**Keywords:** DNA sequencing, Biogeochemistry, Biogeochemistry

## Abstract

Soil microbiomes play important roles in supporting agricultural ecosystems. However, it is still not well-known how soil microbiomes and their functionality respond to fertilization in various cropping systems. Here we examined the effects of 36 years of phosphorus, nitrogen, and manure application on soil bacterial communities, functionality and crop productivity in three contrasting cropping systems (i.e., continuous leguminous alfalfa (AC), continuous winter wheat (WC), and grain-legume rotation of winter wheat + millet - pea - winter wheat (GLR)) in a highland region of China’s Loess Plateau. We showed that long-term fertilization significantly affected soil bacterial communities and that the effects varied with cropping system. Compared with the unfertilized control, fertilization increased soil bacterial richness and diversity in the leguminous AC system, whereas it decreased those in the GLR system. Fertilization, particularly manure application, enlarged the differences in soil bacterial communities among cropping systems. Soil bacterial communities were mostly affected by the soil organic carbon and nitrogen contents in the WC and GLR systems, but by the soil available phosphorous content in the AC system. Crop productivity was closely associated with the abundance of fertilization-responsive taxa in the three cropping systems. Our study highlights that legume and non-legume cropping systems should be disentangled when assessing the responses of soil microbial communities to long-term fertilizer application.

## Introduction

The goal of agriculture is to produce food, pasture, fibers, and energy sources stably and in high volume, while minimizing environmental impacts [1]. The services and processes of agricultural ecosystems largely depend on soil microbiota, which influence nutrient cycling, organic matter decomposition, and plant health and disease [2, 3]. Manipulating microbiota or introducing beneficial microorganisms to construct a simplified synthetic community can significantly improve fertilizer use efficiency and crop disease resistance, thereby increasing crop yield and decreasing environmental impacts [4]. While the contributions of soil microbiota to ecosystem functionality [5] and services [6] have been widely acknowledged, it is still unclear how soil microbiota influence ecosystem productivity. Such knowledge is particularly important to establish sustainable agriculture with high crop yields and low environmental impacts.

Fertilizer application (i.e., fertilization) is the most commonly used management practice in agroecosystems. The combined application of chemical and organic fertilizers is an effective way to reduce pollution and ecological risks, and boost the productivity of agricultural crops [7, 8], and such effects are mainly regulated by changes in microbial community composition [8–10]. For example, Liu et al. [8]. showed that the combined application of chemical and organic fertilizers increased rice yield by reducing soil acidification, increasing soil urease and catalase activities, improving soil nutrient levels, and influencing soil microbial communities (e.g., increasing the relative abundance of *Bacillus* and *Flavobacterium*). However, the current understanding of the response of soil microbiomes to fertilization remains inconclusive. Although fertilization can increase microbial diversity by increasing soil nutrients [11], overfertilization-induced excessive nutrient or organic fertilizer application-induced enrichment of antibiotics and resistance genes could reduce microbial diversity [12, 13]. Additionally, some studies reported no significant changes in the diversity, composition or activity of soil microbial community after fertilization [14–16].

Such contrasting results are largely due to variations in soil type and climate, which can change the habitats for soil microorganisms [17–19]. Generally, different soil types and climates have distinct microbial communities due to their contrasting physicochemical properties, substrate availability, and vegetation [19], which impact the responses of soil microbial communities and functions to fertilization. For example, a previous study showed that chemical fertilizers significantly changed the bacterial community composition in a black and high-fertility soils (Mollisol) and a red and low-fertility soil (Ultisol) but not in a moderately weathered and developed soil (Inceptisol) [16]. Jiao et al. [18]. reported a strong turnover of the soil microbial community in dryland maize soils along the climatic gradient across the continental scale in eastern China. Additionally, vegetation can lead to differences in root exudates, nutrient uptake capacity, litter quality and enzyme activities, causing variations in soil microbial community composition [20, 21], which further complicates the analysis of fertilization effects on soil microbiomes. Currently, it is still unclear how the effects of fertilization on soil microbiomes vary by cropping system due to the lack of comparative analysis among various cropping systems within the same soil type and climate [10], while such a comparison could effectively eliminate the influence of soil type and climate.

Including various crop species, especially legumes, into a cropping system has been proven to be effective in promoting soil ecological function and sustainable crop yield by improving soil structure [22], increasing nutrient availability [23] and microbial diversity [24], reducing soil-borne diseases [21] and enhancing pollution remediation [25, 26]. Leguminous crops are capable of associating with N fixing microbes and releasing amino acids, organic acids, phenolics, and flavonoids to the surrounding soil, which can directly stimulate microbial growth [10, 27] and nutrient cycling [28]. Generally, continuous cultivation of a single crop might result in a decrease in soil quality due to an imbalance of nutrient elements, depletion in enzymatic activities and beneficial microorganisms, enrichment in pathogenic microorganisms, and accumulation of autotoxins during decomposition of crop residues, which ultimately lead to destabilization in soil microbial communities [29]. In contrast, rotations with various crop species promote belowground nutrient transfer (e.g., soil legacy effects) and increase the quality and quantity of substrates and chemically diverse rhizodeposits, which could enhance the growth of plant growth-promoting microorganisms [21, 27]. Because of the microbial symbiotic N fixation and thus the higher soil N levels in legume cropping systems compared with the non-legume cropping systems, we hypothesized that fertilization has contrasting effects on soil microbiomes and their associations with soil properties between legume and non-legume cropping systems, given the important role of N in regulating soil microbiomes.

To test this hypothesis, we examined the effects of long-term fertilization on soil bacterial communities and functionality in three contrasting cropping systems in a highland region. These cropping systems were continuous alfalfa, continuous winter wheat, and grain-legume rotation systems. Each cropping system received chemical and manure fertilizers for 36 years. We collected soil samples from the 0–20 cm soil layer for the measurement of nutrients and microbial activity. We also measured soil bacterial diversity, composition and co-occurring network complexity by using 16S rRNA gene amplicon sequencing. Associations among soil nutrients, bacterial community and plant biomass or crop yield in each cropping system were further analyzed. We aimed to address the following questions: (1) How does long-term fertilization affect soil microbiomes in legume vs. non-legume cropping systems? (2) Does the association between soil nutrients and microbial community vary between legume and non-legume cropping systems? (3) How are soil nutrients and the microbial community related to the productivity of highland agroecosystems?

## Materials and methods

### Study area

**Fig. 1:**
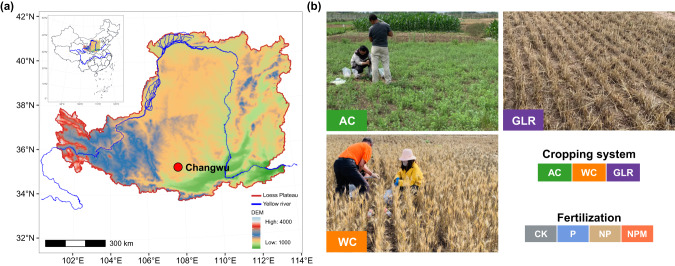
Schematic diagram of the experimental design. **a** Geographic locations of the study sites. **b** Three selected cropping systems in this study. We collected soil samples from a 36-year experiment under diversified cropping systems (i.e., continuous leguminous alfalfa (AC), continuous winter wheat (WC), grain-legume rotation (GLR)) and long-term fertilization (i.e., unfertilized control (CK), phosphorous (P), P and nitrogen (NP) and nitrogen + phosphorus + manure (NPM)) in highland agroecosystems of the Loess Plateau, China.

The long-term fertilization experiment was established in September 1984 in Changwu County, Shaanxi Province (35°12′N, 107°40′E, 1200 m ASL), China (Fig. 1a). The study area is characterized by warm temperate semi-humid continental climate, with a mean annual temperature of 9.2 °C, and a mean frost-free period of 171 days. The mean annual precipitation is 578 mm, with most of the precipitation occurring from July to September. The study site was located in a flat area without any erosion. The soil was classified as a Calcaric Regosol according to the FAO/UNESCO system. The soil texture was clay loam, with contents of >0.02, 0.02–0.002, and <0.002 mm particles of 38%, 38%, and 24%, respectively [30].

### Cropping systems and fertilization experiment design and management

The experiment involved various cropping systems and fertilization treatments (Fig. 1). The cropping systems included a continuous leguminous alfalfa (*Medicago sativa L*.) system (AC), a continuous winter wheat (*Triticum aestivum L*.) system (WC), and a grain-legume rotation (GLR) system with a 3-year planting sequence of winter wheat - millet (*Panicum miliaceum L*.) - pea (*Pisum sativum L*.) - winter wheat [30]. The fertilization treatments included the unfertilized control (CK), phosphorus (P), P and nitrogen (NP), and NP and manure fertilizers (NPM). The WC and GLR systems received all the four fertilization treatments, while the AC system received the CK, P and NPM treatments. The plot (10.3 m × 6.5 m) for each combination of cropping system and fertilization treatment was randomly designed, with three replicates.

The P, N and manure were derived from calcium superphosphate, urea and composted cattle manure (a mixture of 2.3:1 ratio of manure to soils), respectively. The P, N, and manure fertilizers were applied at rates of 26 kg P ha^−1^ yr^−1^, 120 kg N ha^−1^ yr^−1^ and 75 Mg manure ha^−1^ yr^−1^ (dry weight), respectively, for all fertilizer treatments. On a dry weight basis, the manure contained soil organic C (SOC), total N (TN), total P (TP), available N, and available P (OP) contents of 17.68 g kg^−1^, 1.97 g kg^−1^, 0.97 g kg^−1^, 91 mg kg^−1^, and 115 mg kg^−1^, respectively, and the C input from manure was 1.3 Mg ha^−1^ yr^−1^. The NPM treatment supplied 98.8 kg P ha^−1^ yr^−1^, 267.8 kg N ha^−1^ yr^−1^, and 126.8 kg available N ha^−1^ yr^−1^ [30].

The management of the long-term fertilization experiment was the same as that of the local farmland. Fertilizers were applied on the surface before sowing, and then the soil was plowed twice with a cattle-drawn moldboard to a depth of about 20 cm. Wheat and peas were sown in rows 25 cm apart, and alfalfa and millet were broadcast. The winter wheat variety was Changwu 134, the alfalfa and millet were native varieties, and the pea variety was white pea [30]. In 1984, alfalfa was sown by broadcasting at a seeding rate of 7.5 kg ha^−1^, and then fertilizers were surface-applied in mid-April each year since 1984, followed by shallow tillage of the soil with a moldboard plow to a depth of about 10 cm. Alfalfa was harvested for hay in early June and mid-August each year. Winter wheat (187.5 kg ha^−1^), peas (187.5 kg ha^−1^) and millet (62.5 kg ha^−1^) were sown in mid-September, mid-March and early July, respectively. The aboveground biomass (including straw and grains) of winter wheat, peas, and millet was harvested and removed from the subplots in late June, early July, and early October, respectively. The weeds were removed by hand in all cropping systems throughout the growing season. The crop productivities, including aboveground biomass for AC system and grain yield for WC and GLR systems, were measured annually as described previously [30].

### Soil sampling and laboratory analysis of soil properties

In July 2020, we established two subplots (4 m ×5 m) in each plot for soil sampling. In each subplot, soil samples were collected from 0–20 cm depth by randomly taking 5 cores by using a tubular auger (diam. 9.0 cm) and mixing them to make a composite sample. A total of 66 composite soil samples were collected and transported to the laboratory. The soil samples were passed through a 2-mm sieve to remove the roots. There were no stones in the soils. All composite soil samples were divided into four parts: one for the measurement of soil moisture, available N, and potential soil respiration (stored at 4 °C); one for the measurement of soil physicochemical properties (air-dried and stored at room temperature); one for the measurement of soil enzyme activities (stored at −20 °C), and one for microbial community analysis (stored at −80 °C).

The measurement of soil physiochemical properties was performed as described previously [31]. Soil moisture was measured by weighing after oven-drying the soil at 105 °C for 24 h. Soil ammonium (NH_4_^+^) and nitrate (NO_3_^-^) were extracted by shaking the soil with 2 mol L^−1^ KCl and determined by a continuous flow analyzer (Auto-Analyzer-AA3, Seal Analytical, Norderstedt, Germany). The soil SOC and TN concentrations were determined using the dichromate oxidation method and Kjeldahl method, respectively. The soil OP was determined via the Olsen method. The soil pH was measured by a glass electrode meter (InsMark™ IS126, Shanghai, China) at a 1/2.5 soil-water (w/v) suspension.

### Soil enzyme activities and potential soil respiration

The activities of soil enzymes involved in C, N and P acquisition were determined using the microplate-scale fluorometric method [32, 33]. The C-acquisition enzymes analyzed included β−1,4-glucosidase (BG), 1,4-β-D-cellobiohydrolase (CBH) and β-xylosidase (BX). The N-acquisition enzymes analyzed were β−1,4-N-acetylglucosaminidase (NAG) and L-leucine aminopeptidase (LAP), while the P-acquisition enzyme analyzed was alkaline phosphatase (AP). Soil enzyme activities were expressed as nmol g^−1^ dry soil h^−1^. Soil enzyme activities were summed to estimate the total C- acquisition enzymes (EEC = BG + CBH + BX), N- acquisition enzymes (EEN = NAG + CBH), and P- acquisition enzymes (EEP = AP).

The potential soil respiration was determined by using the aerobic incubation method [34]. Before incubation, the moisture content of each of the composite soil samples was adjusted to 60% field moisture capacity. The soil was then acclimatized for one week. Thirty-gram aliquots (*n* = 3) of these soil samples were incubated at 25 °C in a 250-ml screw capped sealed jar in the dark for 30 days. Soil moisture was kept constant by weighing throughout the incubation period. The amount of CO_2_ released from the soil was trapped by 1 mol L^−1^ NaOH solution and measured by titration with 0.5 mol L^−1^ HCl at 1, 3, 7, and 14 days of incubation. The cumulative SOC mineralization (C_min_, mg CO_2_ kg^−1^) was calculated by summing the total amount of CO_2_ released from the soil during the incubation period. The z-score values for the soil enzyme activities (EEC, EEN and EEP) and the potential soil respiration were averaged to characterize soil microbial activity [35, 36].

### DNA extraction, sequencing and bioinformatics analysis

Total soil DNA was extracted from 0.5 g soil by using a FastDNA Spin Kits (MP Biomedicals, Solon, OH, USA) according to the manufacturer’s instructions. The primers 341F (5′-CCTAYGGGRBGCASCAG-3′) and 806 R (5′-GGACTACHVGGGTWTCTAAT-3) were used to amplify the V3-V4 regions of the 16S rRNA gene. The PCR program included an initial denaturation at 98 °C for 1 min, 30 cycles of denaturation at 98 °C for 10 s, annealing at 50 °C for 30 s, and extension at 72 °C for 30 s, followed by a final extension at 72 °C for 5 min. The amplified products were purified and tagged with Illumina sequencing adaptors by using a Qiagen Gel Extraction Kit (Qiagen, Germany), The tagged amplicons were purified, quantified, pooled and sequenced on an Illumina NovaSeq platform with 250-bp pair-end sequencing at the Novogene Company, Beijing, China.

Sequences were trimmed, quality filtered, demultiplexed, and used to identify amplicon sequence variants (ASVs) by using the DADA2 program [37] on the QIIME2-2020.11 platform [38]. We only used the forward reads that were truncated at position 220 bp for bioinformatics analysis, as the low sequencing quality near the 3’ end of the reads would greatly reduce the number of successfully assembled reads. The SILVA 138 database and Naïve Bayes classifier were used for taxonomic classification. We subsequently removed singletons, chloroplast and mitochondrial sequences to obtain a total of 4,182,288 high-quality sequences (range 38,463–87,256; median 64,092 sequences per sample). Rarefaction curves indicated that the sequencing depth had reached saturation (Supplementary Fig. S1). The ASV feature table was rarefied to minimum sequences (i.e., 38463) across all samples for downstream analysis.

### Statistical analysis

All statistical analyses were performed in R language version 4.1.0 (v4.1.0; http://www.r-project.org/). Differences in soil properties, α-diversity, microbial activities and crop productivity were assessed by linear mixed effect models, where cropping system and fertilization regime were included as fixed effects, and replicate plot was considered as a random effect using lme4 and lmerTest packages [39]. The assumptions of homoscedasticity and normality of the residuals were examined via the Shapiro–Wilk test and residual versus fitted plots, respectively. When these assumptions were not satisfied, the data were log or square root transformed. The Wilcox rank sum test was used to determine significant effects at the *P* < 0.05 level. Venn diagrams were generated to show the numbers of shared and unique ASVs. Principal coordinate analysis (PCoA) based on Bray–Curtis distance and permutational multivariate analysis of variance (PERMANOVA) were performed using the vegan package [40] to assess the variations in bacterial communities. The distance-based redundancy analyses (dbRDA) were used to assess the relationship between the bacterial communities and soil properties.

Differential abundance analyses were performed, using the *DESeq2* negative-binomial Wald test [41] and Benjamini Hochberg’s correction [42], to identify the ASVs with increased or decreased relative abundance under the fertilization conditions (i.e., fertilization-responsive/sensitive ASVs) for each cropping system. Similarly, ASVs with increased or decreased relative abundances under specific cropping system were identified for each fertilization regime. We also identified the core taxa present in bacterial communities across all cropping systems and fertilization treatments. The ASVs that occurred in all samples (taxa with an occurrence prevalence of 100%) were defined as the core taxa [43], and they might also represent potentially important players present in all cropping systems and fertilization treatments.

Co-occurring bacterial networks for each cropping system were constructed to infer potential associations of the bacterial community using the program Sparse Correlations for Compositional data (SparCC) [44], considering that the amplicon-based datasets were compositional (zero-inflated data). Taxa with an average relative abundance greater than 0.01% were used for correlation calculation. The correlations with *r* > 0.65 and false discovery rate <0.05 were used for network construction. The 1000 Erdӧs-Réyni random networks were constructed for comparison with the real network. Furthermore, the network properties of each sample were calculated using the subgraph function in the igraph package [45]. The Wilcox test was used to determine significant differences among treatments at the *P* < 0.05 level. Network robustness was evaluated by normalized natural connectivity. Although correlation networks do not necessarily represent the real biological interactions between species, they can provide valuable insights into species co-occurrence patterns and elucidate the mechanisms driving their community assembly [46–48].

Mantel analysis was used to explore the relationships among soil properties, microbial activity, crop productivity and bacterial communities based on all, core and fertilization-responsive datasets by using the linkET package (https://github.com/Hy4m/linkET). Random forest and variance partitioning analyses were also done to evaluate the relative effects of soil properties, and core and fertilization-responsive communities on microbial activity and crop productivity by using the rfPermute packages [49] and the vegan package. The tuneRF function was used to train a series of models to detect the optimal mtry values (number of variables randomly sampled as candidates at each split), which were used to re-run the subsequent forest models. The tuneRF function is a specific utility to tune the mtry parameter based on out-of-bag error [50, 51].

Piecewise structural equation models (SEMs) were constructed to examine the direct and indirect effects of changes in soil nutrients (=significant outputs from the random forest analysis) and core or responsive communities on microbial activity and crop productivity after long-term fertilization in each cropping system. Each piecewise model was constructed using a linear mixed-effects model with replicate plots as random effects. The microbial activity data were log-transformed to meet the normality assumption and all the variables were standardized for the effects to be directly comparable [52]. The full models containing all potential paths of the prior models were tested (Supplementary Fig. S2). The models were modified by removing insignificant direct and indirect paths when the initial models did not produce an adequate fit. Shipley’s d-separation test was used to examine whether any paths were missing from the model [53]. We reported the standardized coefficient for each path from each component model, and the Fisher’s C statistics, AIC and BIC values, conditional R^2^ (R_c_^2^) and marginal R^2^ (R_m_^2^) of the overall model obtained by using the piecewiseSEM package [54].

## Results

### Soil properties, microbial activity and crop productivity

Cropping system significantly influenced most soil physicochemical properties and microbial activity (*P* < 0.05, Table 1). The AC system had the highest SOC, TN, C/N, NH_4_^+^, NO_3_^−^, EEC, EEN, EEP and microbial activity but the lowest OP averaged across fertilization treatments (Table 1). In addition, wheat yield was significantly higher in the GLR system than in the WC system (Supplementary Fig. S3).

**Table 1:** Effects of fertilization and cropping system on soil properties, microbial activity and crop productivity.

Cropping system	Fertilization	SOC (g kg^−1^)	TN (g kg^−1^)	C/N (unitless)	OP (mg kg^−1^)	NH_4_^+^ (mg kg^−1^)	NO_3_^−^ (mg kg^−1^)	N/P (unitless)	SM (%)	pH (unitless)	MA (unitless)	Productivity (Mg ha^−1^)
AC	CK	11.84 ± 1.63 Aa	1.54 ± 0.20 Aa	7.64 ± 0.27 Aa	2.63 ± 0.38 Bb	2.26 ± 0.14 Aa	24.64 ± 2.04 Aab	11.08 ± 1.28 Aa	18.28 ± 0.64 Aa	8.00 ± 0.16 Aa	0.34 ± 0.44 Aa	8.11 ± 0.05 c
	P	13.39 ± 1.73 Aa	1.66 ± 0.19 Aa	7.98 ± 0.15 Aa	10.52 ± 2.55 Ba	1.90 ± 0.18 Aa	16.64 ± 2.64 Ab	2.21 ± 0.44 Ab	18.17 ± 0.77 Aa	8.03 ± 0.13 Aa	0.92 ± 0.55 Aa	9.43 ± 0.00 b
	NPM	14.31 ± 1.09 Aa	1.76 ± 0.11 Aa	8.11 ± 0.12 Aa	15.11 ± 4.27 Ba	1.84 ± 0.08 Aa	25.84 ± 1.22 Aa	2.61 ± 0.57 Ab	19.88 ± 0.78 Aa	8.10 ± 0.06 Aa	0.70 ± 0.29 Aa	12.45 ± 0.07 a
WC	CK	7.32 ± 0.21 Bb	1.04 ± 0.05 Bb	7.09 ± 0.17 Abc	4.45 ± 0.39 Ab	1.7 ± 0.18 Aa	2.99 ± 0.68 Bc	1.10 ± 0.17 Ca	19.04 ± 0.43 Aab	8.24 ± 0.06 Aab	−0.74 ± 0.13 Bc	1.61 ± 0.01 c
	P	7.42 ± 0.29 Bb	0.98 ± 0.04 Bb	7.58 ± 0.09 Ba	25.83 ± 5.06 Aa	2.21 ± 0.18 Aa	2.31 ± 0.39 Cc	0.22 ± 0.05 Cb	18.26 ± 0.45 Ab	8.31 ± 0.04 Aab	−0.11 ± 0.08 Ab	1.43 ± 0.01 d
	NP	8.22 ± 0.60 Ab	1.12 ± 0.04 Ab	7.40 ± 0.56 Aab	8.67 ± 2.68 Ab	2.05 ± 0.16 Aa	6.09 ± 0.34 Bb	1.38 ± 0.32 Aa	18.08 ± 0.55 Ab	8.34 ± 0.02 Aa	0.07 ± 0.06 Aab	5.18 ± 0.01 b
	NPM	10.35 ± 0.23 Ba	1.46 ± 0.04 Ba	7.10 ± 0.12 Bbc	34.06 ± 6.37 Aa	1.93 ± 0.16 Aa	18.83 ± 0.99 Ba	0.74 ± 0.16 Ba	20.21 ± 0.68 Aa	8.13 ± 0.08 Ab	0.21 ± 0.11 ABa	5.55 ± 0.01 a
GLR	CK	6.62 ± 0.34 Bb	0.91 ± 0.03 Cd	7.27 ± 0.19 Aab	1.93 ± 0.30 Bc	2.19 ± 0.13 Aa	3.95 ± 0.61 Bc	3.78 ± 0.94 Ba	18.79 ± 0.39 Aa	8.30 ± 0.12 Aa	−0.42 ± 0.19 Ab	1.03 ± 0.03 d
	P	7.39 ± 0.32 Bb	1.04 ± 0.04 Bc	7.08 ± 0.18 Cb	22.49 ± 3.56 Aa	2.58 ± 0.40 Aa	5.67 ± 0.67 Bc	0.42 ± 0.07 Bd	19.02 ± 0.44 Aa	8.14 ± 0.12 Aa	−0.15 ± 0.25 Aab	1.25 ± 0.00 c
	NP	9.87 ± 0.46 Aa	1.24 ± 0.03 Ab	7.93 ± 0.31 Aa	11.16 ± 1.81 Ab	1.61 ± 0.13 Bb	12.43 ± 0.56 Ab	1.40 ± 0.18 Ab	19.11 ± 0.16 Aa	8.04 ± 0.09 Ba	−0.05 ± 0.18 Aab	3.08 ± 0.01 b
	NPM	10.37 ± 0.77 Ba	1.52 ± 0.06 ABa	6.82 ± 0.40 Bb	33.64 ± 4.29 Aa	2.18 ± 0.23 Aab	20.18 ± 2.23 Ba	0.70 ± 0.09 Bc	19.17 ± 0.4 Aa	8.21 ± 0.07 Aa	−0.02 ± 0.17 Ba	3.42 ± 0.00 a

Each value represents the mean ± standard error (*n* = 6). The uppercase letters indicate significant differences among different cropping systems under the same fertilization treatment, whereas the lowercase letters indicate significant differences among different fertilization treatments under the same cropping system. The differences in crop productivity among the different cropping systems are not examined because the crop productivity is actually the aboveground biomass of alfalfa in the AC system, while it represents the grain yield in WC and GLR systems. Significant differences were assessed by Wilcox rank sum test (*P* < 0.05).

*AC* continuous alfalfa system, *WC* continuous wheat system, *GLR* grain-legume rotation system, *CK* unfertilized control, *P* phosphorus, *NP* nitrogen + phosphorus, *NPM* nitrogen + phosphorus + manure, *SOC* soil organic carbon, *TN* total nitrogen, *C/N* ratio of carbon to nitrogen, *OP* available phosphorus, *NH*_*4*_^*+*^ ammonium, *NO*_*3*_^*−*^ nitrate, *N/P* ratio of mineral nitrogen to available phosphorus, *SM* soil moisture, *pH* soil pH (H_2_O), *MA* soil microbial activity (z-score of enzyme activities and OC mineralization), Productivity, crop productivity.

The P treatment increased OP but had minimum effects on the other soil properties in the three systems. Additionally, the P treatment increased crop productivity in the AC and GLR systems but decreased it in the WC system. The NPM treatment significantly increased soil nutrients (SOC, TN, NO_3_^−^), EEP, microbial activity, and crop productivity (AC, WC and GLR). Additionally, such increases were greater in the WC and GLR systems than the AC system (Table 1).

In our experiment, only the WC and GLR systems received the NP treatment. In both systems, the NP treatment significantly increased soil available N and P and microbial activity but had minimum effects on the other soil properties.

### Soil bacterial community diversity and composition

**Fig. 2:**
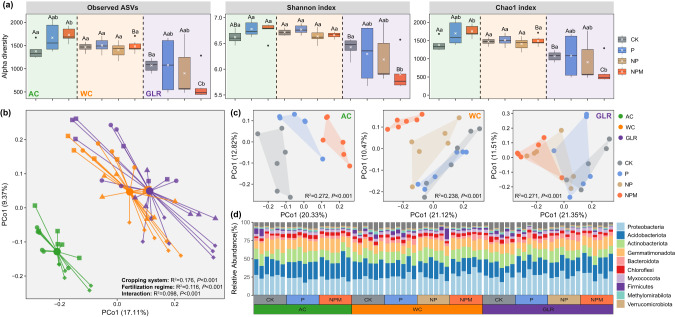
Variation in soil bacterial communities induced by cropping system and long-term fertilization. **a** Bacterial species richness and diversity as estimated by the Observed, Shannon and Chao1 indexs. **b** Principal coordinates analysis (PCoA) plot based on Bray–Curtis distance as affected by cropping system, fertilization and their interactions. **c** PCoA plot based on Bray–Curtis distance as affected by fertilization in each cropping system. **d** The relative abundances (%) of major taxonomic groups at the bacterial phyla level. Legend: AC continuous alfalfa system, WC continuous wheat system, GLR grain-legume rotation system, CK unfertilized control, P phosphorus, NP nitrogen + phosphorus, NPM nitrogen + phosphorus + manure. The uppercase letters indicate significant differences among different cropping systems under the same fertilization treatment, whereas the lowercase letters indicate significant differences among different fertilization treatments under the same cropping system. Significant differences were assessed by the Wilcox rank sum test at the *p* < 0.05 level.

The observed number of ASVs and Shannon and Chao1 index values were significantly larger in the AC and WC soils than in the GLR soils when comparing CK and NPM treatments (Fig. 2a). Additionally, when examined either across or within fertilization treatments, the number of unique bacterial ASVs in soils was always largest in the AC system and lowest in the GLR system (Supplementary Fig. S4a).

The effects of fertilization on soil microbial diversity varied significantly by cropping system. While the observed number of ASVs, Shannon and Chao1 index values were not different by fertilization in the WC system, these values generally increased and decreased in the AC and GLR systems, respectively (Fig. 2a). The effect of the NPM treatment on microbial diversity was greater than that of the P and NP treatments in all three cropping systems. Similarly, the number of unique ASVs was the largest in the NPM treatment in the AC and WC systems, but was the lowest in the NPM treatment in the GLR system (Supplementary Fig. S4b).

The ASVs in the studied soils were assigned to 38 unique phyla. On average, *Proteobacteria* (25.9%), *Acidobacteriota* (21.5%), *Actinobacteriota* (13.9%), *Gemmatimonadota* (13.6%) and *Bacteroidota* (4.4%) accounted for more than 79% of the total sequence reads (Fig. 2). Principal coordinates (PCoA) and PerMANOVA analyses showed that soil bacterial communities were significantly different by cropping system (*R*^2^ = 0.191, *P* < 0.001), fertilization (*R*^2^ = 0.106, *P* < 0.001) and their interaction (*R*^2^ = 0.089, *P* < 0.001) (Fig. 2b). In each cropping system, soil bacterial communities varied significantly by fertilization treatment (*R*^2^ = 0.238–0.272, *P* < 0.001, Fig. 2c, Supplementary Tables S1 and S2).

### Variations in the core and fertilization-responsive taxa among cropping systems

**Fig. 3:**
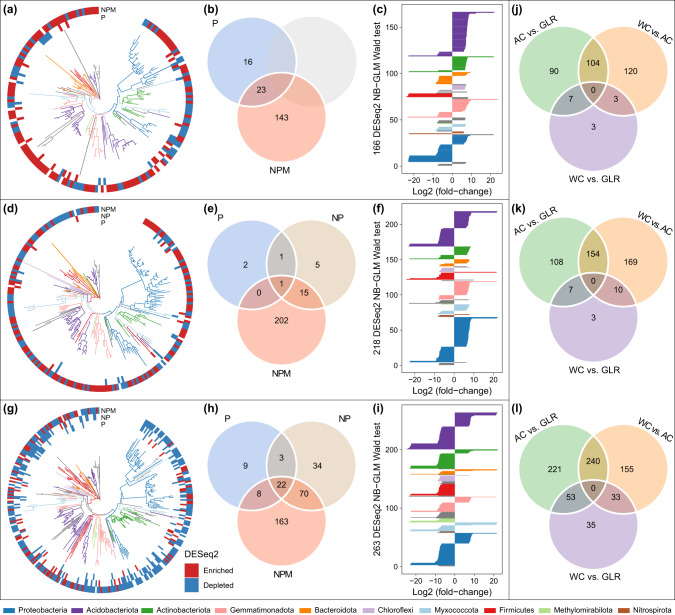
Responses of microbes to fertilization in each cropping system or to cropping system under each fertilization treatment. The number of differentially abundant ASVs was identified using the *DESeq2* package (FDR < 0.05, Supplementary Table S3) using the unfertilized treatment as a control in the AC (**a**–**c**), WC (**d**–**f**) and GLR (**g**–**i**) systems, or using a specific cropping system as a control under the CK (**j**), P (**k**) and NPM (**l**) treatments. “Enriched” and “depleted” ASVs indicated that they were more and less abundant, respectively, in the specific fertilization treatment than in the unfertilized treatment. The three figures from left to right (i.e., **a**–**c**) showed the phylogenetic tree of fertilization-sensitive ASVs, the number of shared/unique fertilization-sensitive ASVs among different fertilization treatments, and the taxonomic attributes of sensitive ASVs responding to NPM treatment, respectively. Legend: AC continuous alfalfa system, WC continuous wheat system, GLR grain-legume rotation system, CK unfertilized control, P phosphorus, NPM nitrogen + phosphorus + manure. Detailed results are presented in Supplementary Table S3.

Long-term fertilization did not affect the relative abundance of the 24 core taxa in the three cropping systems (Supplementary Fig. S5). The number of fertilization-responsive ASVs, identified by the *DESeq2* analysis using the control (CK) treatment as the reference, was smaller in the P and NP treatments (4–129) as compared with the NPM treatment (166–263) (Fig. 3). However, the effects of fertilization on the relative abundance of these ASVs varied by cropping system. For the AC system, 20 and 106 ASVs were enriched in the P and NPM treatments, whereas, 19 and 60 ASVs were depleted, when compared with CK. For the WC system, 1, 9 and 120 ASVs were enriched in the P, NP and NPM treatments, respectively, whereas, 3, 13 and 98 ASVs were depleted. For the GLR system, 10, 31 and 68 ASVs were enriched in the P, NP and NPM treatments, whereas, 32, 98 and 195 ASVs were depleted. Such variations among cropping systems were particularly true for the NPM treatment (Fig. 3a–i). In addition, the numbers of unique and shared crop system-responsive ASVs increased from CK to P to NPM treatment (Fig. 3j–l).

The fertilization-responsive taxa were mainly affiliated with the phyla *Proteobacteria*, *Acidobacteriota*, *Actinobacteriota*, *Gemmatimonadota* and *Firmicutes* (Fig. 3). However, such fertilization-responsive taxa were cropping-system dependent. In the AC system, taxa whose relative abundance at the family level increased in the NPM treatment (i.e., NPM-enriched taxa) included *Pyrinomonadaceae*, *Gemmatimonadaceae*, *Enterobacteriaceae*, *Frankiaceae*, *Nitrospiraceae* and *Solirubrobacteraceae*, whereas those taxa that decreased in abundance in the NPM treatment (i.e., NPM-depleted taxa) included *Comamonadaceae*, *Lachnospiraceae* and *Haliangiaceae* (Supplementary Tables S3 and S4). In the WC system, NPM-enriched taxa included the families *Xanthobacteraceae, Xanthomonadaceae*, *Hymenobacteraceae*, *Rhodospirillaceae, Rhodobacteraceae, Oxalobacteraceae*, *Rhizobiaceae*, and *Nocardioidaceae*, whereas NPM-depleted taxa included *Acidiferrobacteraceae*, *Lachnospiraceae, Roseiflexaceae, Solibacteraceae* and *Coriobacteriaceae* (Supplementary Tables S3 and S4). In the GLR system, NPM-enriched taxa included *BIrii41, Microscillaceae, Rhizobiaceae, S0134_terrestrial_group, BD2–11_terrestrial_group* and *Streptomycetaceae*, whereas NPM-depleted taxa included *Clostridiaceae, Erysipelotrichaceae, Haliangiaceae, Helicobacteraceae, Latescibacterota, Phormidiaceae, Rhodobacteraceae, Rokubacteriales*, and *Solirubrobacteraceae* (Supplementary Tables S3 and S4).

### Bacterial co-occurrence network patterns

**Fig. 4:**
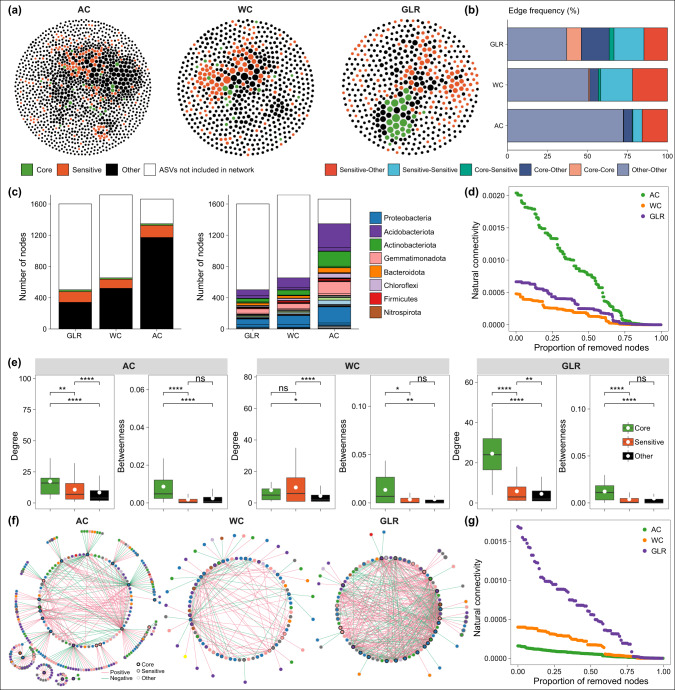
Bacterial co-occurrence networks. **a** The bacterial co-occurrence networks in the AC, WC and GLR systems. Nodes represent individual ASVs; the size of each node is proportional to the degree of the node; nodes were colored according to core, fertilization-sensitive and other taxa. **b** Frequency of connections among the three types of nodes in each network. **c** The proportion and taxonomy of core, fertilization-sensitive and other nodes included in each co-occurrence network. Portion of nodes not included in the network is also reported (white portion of bars). **d** Co-occurrence network robustness was represented by normalized natural connectivity. **e** Node-level topological features of core, sensitive and other taxa, including the degree and betweenness centrality in each network. **f** The core network, which was the relationships between core ASVs and the ASVs they recruited (nodes connected to the core ASVs) in each cropping system. **g** Core network robustness was represented by normalized natural connectivity. Legend: AC continuous alfalfa system, WC continuous wheat system, GLR grain-legume rotation system. Asterisks denote significant differences based on the Wilcoxon rank sum tests. *p < 0.05, **p < 0.01 and, ***p < 0.001.

The degree of nodes for the co-occurring network in each cropping system exhibited a power-law distribution, indicating scale-free and non-random distribution patterns (Supplementary Fig. S6). The AC system had a more complex and connected network than the GLR and WC systems (Fig. 4a–c and Supplementary Fig. S7). Moreover, the network robustness was greater in the AC system than in the GLR and WC systems (Fig. 4d).

The variations in the parameters of the co-occurring network among fertilization treatments were also cropping-system dependent (Supplementary Fig. S7). For example, fertilization significantly increased the number of nodes and edges, degree of co-occurring network in the AC system; whereas it did not significantly influence these network properties in the WC and GLR systems (Supplementary Fig. S7). Moreover, such effects were greater in the NPM treatment than in the P and NP treatments.

The core and responsive nodes had significantly larger degrees and betweenness centrality than the other nodes in the three cropping systems (Fig. 4e). Moreover, the co-occurring network in the GLR system had more connections to the core taxa (core-core, core-responsive, and core-other taxa) than that in the AC system (Fig. 4b). The network of core taxa was more complex and robust in the GLR system than in the WC and AC systems (Fig. 4f, g). These results suggest that the core and responsive taxa play important roles in maintaining the association and robustness of soil bacterial communities, although such roles vary by cropping system.

### Relationship among soil properties, bacterial communities, microbial activities, and crop productivity

**Fig. 5:**
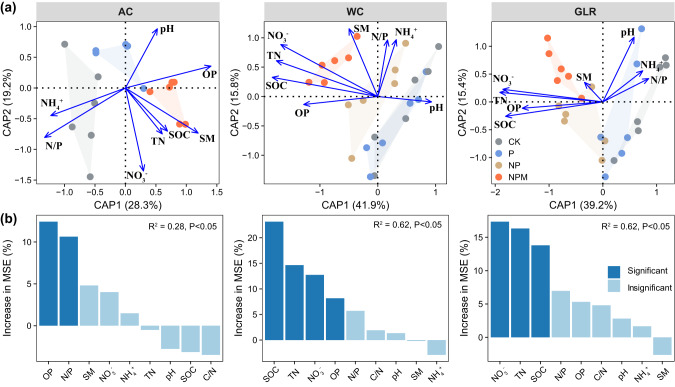
Changes in soil bacterial communities driven by soil physicochemical properties. **a** Relationships between the bacterial community composition and soil properties in each cropping system as assessed by distance-based redundancy analyses (dbRDA) based on Bray–Curtis dissimilarity. Sample points are colored according to the different fertilization treatments. **b** Random forest showing the relative importance of soil properties in predicting the bacterial community composition (first axis of principal coordinates analysis) in each cropping system. The predictor importance is reported by the percentage increase in mean square error (MSE). Legend: AC continuous alfalfa system, WC continuous wheat system, GLR grain-legume rotation system, CK unfertilized control, P phosphorus, NP nitrogen + phosphorus, NPM nitrogen + phosphorus + manure, SOC soil organic carbon, TN total nitrogen, C/N ratio of carbon to nitrogen, OP available phosphorus, NH_4_^+^ ammonium, NO_3_^−^ nitrate, N/P ratio of mineral nitrogen to available phosphorus, SM soil moisture, pH soil pH (H_2_O).

**Fig. 6:**
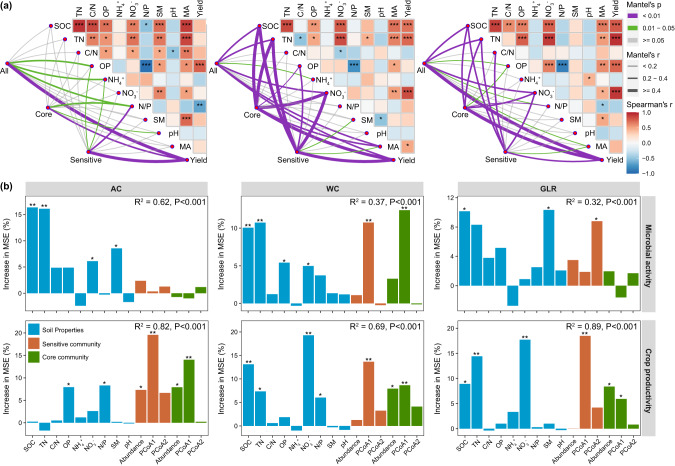
Linking soil physicochemical properties, and bacterial communities to microbial activity and crop productivity in each cropping system. **a** Mantel test examining the relationship between soil properties, microbial activity, crop productivity and the compositions of all, core and fertilization-sensitive communities (PCoA1 and PCoA2, first and second axis of principal coordinates analysis) in each cropping system. **b** Random forest showing the relative importance of soil properties, compositions of core and fertilization-sensitive communities (PCoA1 and PCoA2) in predicting the microbial activity and crop productivity. The predictor importance is reported by the percentage increase in mean square error (MSE). Legend: AC continuous alfalfa system, WC continuous wheat system, GLR grain-legume rotation system, SOC soil organic carbon, TN total nitrogen, C/N ratio of carbon to nitrogen, OP available phosphorus, NH_4_^+^ ammonium, NO_3_^−^ nitrate, N/P ratio of mineral nitrogen to available phosphorus, SM soil moisture, pH soil pH (H_2_O), Abundance cumulative relative abundance core or sensitive taxa, PCoA1 and PCoA2 first and second axis of principal coordinates analysis based on core or fertilization-sensitive community.

The dbRDA and random forest analyses showed that soil bacterial communities were mostly affected by soil OP and N/P in the AC system, but by SOC, TN and NO_3_^-^ in the WC and GLR systems (Fig. 5). Moreover, soil properties (e.g., SOC, TN, NO_3_^-^, OP), core and responsive communities (e.g., first components of PCoA and relative abundance) contributed significantly to predicting soil microbial activities and crop productivity in each cropping system (Fig. 6 and Supplementary Figs. S8 and S9). For example, microbial activities and crop productivity were positively correlated with the relative abundance of responsive taxa in the AC (e.g., *Pyrinomonadaceae*, *Gemmatimonadaceae*, *Enterobacteriaceae*, *Frankiaceae* and *Solirubrobacteraceae*), WC (e.g., *Xanthomonadaceae*, *Xanthobacteraceae*, *Hymenobacteraceae*, *Rhodospirillaceae*, *Rhodobacteraceae*, *Oxalobacteraceae*, *Rhizobiaceae* and *Nocardioidaceae*) and GLR (e.g., *BIrii41*, *Microscillaceae*, *Rhizobiaceae* and *Streptomycetaceae*) systems (Supplementary Table S3 and Supplementary Fig. S9). Therefore, core and responsive taxa are important in maintaining soil microbial activities as well as crop productivity in the highland agroecosystem studied.

**Fig. 7:**
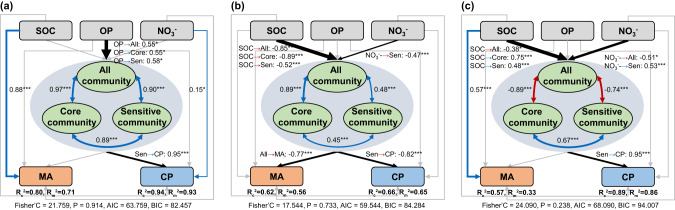
Linkages between soil bacterial communities and microbial activity or crop productivity in various cropping systems. Direct and indirect drivers of soil microbial activity and crop productivity in the AC (**a**), WC (**b**) and GLR (**c**) systems. The piecewise structural equation model assessing the direct and indirect effects of soil properties and bacterial community composition (first axis of principal coordinates analysis) on soil microbial activity and crop productivity in three cropping systems. Numbers adjacent to arrows show the standardized path coefficients. The conditional R^2^ (R_c_^2^) denotes the variance explained by both fixed and random effects of ‘sampling plot’, whereas the marginal R^2^ (R_m_^2^) denotes the variance explained by fixed effects. Blue and red arrows represented the significant positive paths and negative paths, respectively. Legend: SOC soil organic carbon, OP available phosphorus, NO_3_^−^, nitrate; MA microbial activity, CP crop productivity. Asterisks denote significant paths. *p < 0.05, **p < 0.01, and ***p < 0.001.

We further used SEM to clarify the direct and indirect effects of soil nutrients on microbial activities and crop productivity (Fig. 7). Soil microbial communities were affected by OP in the AC system, but by SOC and NO_3_^−^ in the WC and GLR systems. Soil microbial activities were directly influenced by SOC in the AC and GLR systems, and indirectly influenced by SOC by altering microbial communities in the WC system. The crop productivity in the AC system was affected directly by NO_3_^-^ and indirectly by OP through the changes in microbial communities (i.e., responsive communities). The crop productivity in the WC and GLR systems was only indirectly affected by SOC and NO_3_^−^ through the changes in microbial communities. These results highlighted the important role of fertilization-responsive communities in regulating the effects of fertilization on crop productivity in these highland agroecosystems.

## Discussion

### Fertilization influences soil nutrients and microbial activities

Fertilization treatments such as NP and NPM significantly increased soil nutrients and microbial activities in our study. This is likely because of the input of exogenous nutrients, as previously suggested [55]. Long-term application of fertilizers, particularly organic fertilizers can significantly increase soil nutrient pools [7, 8], which stimulate the activities of microbial communities [8, 56]. In addition, fertilizer amendment could activate soil microbes that can respond quickly to the added substrates [9], which would further increase soil microbial activities.

We also found that the effects of NPM on soil nutrients were greater in the WC and GLR systems than in the AC system. This is probably because the microbial symbiotic N fixation in the leguminous AC system resulted in a relatively higher soil N level and microbial activities than those in the other cropping systems even when fertilizers were not applied [26, 57]. Such high soil N levels and microbial activities in the unfertilized control plot could lower the positive effect of fertilization in the leguminous AC system. Therefore, variations in cropping systems should be considered when evaluating the impact of fertilization on agricultural soils [25, 30]. Our results also suggested that alfalfa (leguminous crop) can function as a biofertilizer, similar to a previous study [58], which is of great significance for sustainable agriculture, particularly considering the high costs of manure management.

### The responses of soil bacterial communities to fertilization vary by cropping system

We found that the response of soil bacterial communities to fertilization differed between legume and non-legume cropping systems, supporting our hypothesis. Fertilization, particularly the NPM treatment, significantly increased soil bacterial diversity in the AC system in our study. This is probably because the combination of fertilization and leguminous alfalfa can provide various niches for microbes and thus increase bacterial α-diversity [59, 60]. In our study, we observed more enriched ASVs (106) than depleted ASVs (60) after the NPM treatment in the AC system (Fig. 3b). Similar to this study, Ye et al. [61]. reported that manure fertilizer significantly increased the α-diversity of soil bacteria in a monoculture leguminous peanut system in southern China. In contrast to the AC system, fertilization did not affect the bacterial α-diversity in the WC systems in our study. The numbers of enriched and depleted ASVs were also similar (120 and 98, respectively) after fertilization in the WC system. Similar to our study, no difference in the bacterial α-diversity between fertilized and unfertilized soils was recently reported in continuous wheat [15], tobacco [17] and rice systems [8]. Contrary to the AC and WC systems, the α-diversity of soil bacteria in the GLR system decreased in response to the fertilization treatments in our study. More ASVs were depleted (195) than enriched (68) by fertilization in the GLR system (Fig. 3h). Similar to this study, reduced bacterial α-diversity was previously reported in soils with frequent crop rotations including peas [62–64], although it is still unclear why this happened.

We found that fertilization increased the number of crop system-responsive ASVs (Fig. 3j–l). This is probably related to the positive effects of fertilization on plant growth. Plants under fertilization treatments grew better and therefore could have larger impacts on soil microbiota than those under unfertilized or less fertilized treatments. In our long-term experiment, the NP and NPM treatments significantly increased the aboveground (Table 1) and belowground biomass [30] in each of the cropping systems. The increase in these biomasses have significant impacts on soil organisms [65, 66], mainly by the return of litters, roots into the soil, and by the rhizodeposition such as phenolic exudates, root cells and mucilage, which altered soil physicochemical properties and thus affect the microbial community composition [21].

### Bacterial co-occurrence network patterns vary by fertilization and cropping system

Network complexity (nodes, edges, and degrees) and robustness were higher in the AC system than in the other systems in our study. This is probably related to the higher nutrient pools in the legume system, as previously suggested [59]. We also observed positive correlations between soil nutrients and network parameters (Supplementary Fig. S10). Generally, nitrogen-rich compounds produced by legumes are more easily decomposed by soil microorganisms, which can influence biological activities at higher trophic levels, including increased complexity of food webs and the resistance to ecosystem disturbance [67]. Additionally, higher bacterial diversity in the AC system might have also contributed to the high network complexity and robustness observed in this highland agroecosystem (Fig. 2a), similar to other studies performed in dryland plantation [59] and manipulated experiment on soil biodiversity loss [5].

The bacterial networks in the GLR system, which included leguminous pea as one of the rotation crops, were also more complex (i.e., with higher connectivity and robustness) than those in the WC system (Fig. 4d and Supplementary Fig. S7). Robust bacterial communities are closely associated with core taxa that play a major role in maintaining bacterial community associations even under disturbance [68, 69]. The GLR system received more intensified disturbances than the other systems, including more frequent tillage and crop rotations as well as a shorter duration of crop cover [30]. Given that core taxa can occupy a wide range of ecological niches and contribute to stabilizing microbial communities after perturbation [69, 70], the role of the core communities in maintaining the robust bacterial communities was likely more important in the GLR system than in the WC system.

The NPM treatment increased the network complexity (nodes, edges and degree) in the AC system in this study. This is probably because of the enhanced soil nutrients and α-diversity [5, 68]. However, the NPM treatment did not affect the network complexity in the WC and GLR systems (Supplementary Fig. S7). Decreased bacterial diversity, which was seen in the GLR system with the NPM treatment, can reduce potential microbial connections [5, 61]. Moreover, the nutrient accumulation induced by the NPM treatment can provide niches for microbes and increase potential microbial connections. These negative and positive effects of the NPM treatments on network complexity may have canceled each other out, resulting in a neutral effect of NPM on the network complexity in the WC and GLR systems.

### Association of the soil bacterial community with soil properties varies by cropping system

Our results showed that the effects of soil properties on the bacterial community varied by cropping system. In the AC system, soil OP and N/P influenced bacterial communities, while in the WC and GLR systems, SOC, TN and NO_3_^-^ influenced bacterial communities (Fig. 5b). In general, N-fixing legumes require more P than non-N-fixing plants [67]; therefore, the soils in the AC system are likely more susceptible to P stress [30]. In our experiment, soil OP was significantly lower in the AC system than in the WC and GLR systems, even with the NPM treatment (Table 1). Nitrogen fixation by alfalfa can also increase SOC and N as seen in our study (Table 1) and other studies [30, 57], which could alleviate the N stress for soil microbiota. Therefore, soil bacterial communities in the alfalfa cropping system (i.e., the AC system) can be more influenced by P than by SOC and N, while those in non-alfalfa cropping systems (i.e., the WC and GLR systems) are more influenced by N than by P, as evidenced by the higher OP and lower SOC and N in the WC and GLR systems than in the AC system (Table 1). This can explain why SOC and N were identified as the factors influencing soil bacterial communities in the WC and GLR systems (Fig. 5). Although the GLR system included leguminous pea, this leguminous crop was grown for only approximately 4 months of the 3-year rotation, and our sampling was conducted 2 years after pea cultivation, therefore the effects of pea might be minimal.

### Linking fertilization-sensitive taxa to crop productivity

We found significant associations between fertilization-responsive taxa and crop productivity in all three cropping systems (Fig. 6a). The effects of fertilization on soil microbial activity and crop productivity were attributed not only to the increase in soil nutrients, but also to the shaping of the microbial communities by the enrichment of specific taxa (i.e., fertilization-responsive taxa) similar to previous studies [8, 71]. These responsive taxa were likely active in soil environments and may play important roles in maintaining crop productivity [8, 72]. Optimizing the spatiotemporal dynamics of responsive taxa is thus important for sustainable agroecosystems with high resource efficiency and high resistance or resilience to disturbance [8, 72].

Although the fertilization-responsive taxa varied by cropping system, many of them are known to play important roles in maintaining crop productivity. For example, fertilization-enriched taxa in the AC system included *Pyrinomonadaceae* and *Gemmatimonadaceae*, some of which can hydrolyze complex polymers [73, 74], *Enterobacteriaceae* and *Frankiaceae*, some of which can degrade glucose-derived C and fix atmospheric N [75–78], and *Solirubrobacteraceae*, some of which can suppress plant pathogens [79]. In the WC system, fertilization-enriched taxa included *Sphingomonadaceae*, *Xanthomonadaceae*, *Rhodobacteraceae*, and *Nocardioidaceae*, some of which can degrade various complex compounds and pollutants [80–85], and *Rhodospirillaceae*, *Xanthobacteraceae*, and *Oxalobacteraceae*, some of which can fix N [86–88]. In the GLR system, fertilization-enriched taxa included *BIrii41* and *Streptomycetaceae*, some of which are known to be resistant to both heavy metals and pathogens [83, 89, 90], *Microscillaceae* and *Streptomycetaceae*, some of which can degrade gelatin and plant residues [55, 91], and *Rhizobiaceae*, some of which can fix N and solubilize inorganic P [75, 90]. Most of these fertilization-enriched taxa have also been reported to have the ability to improve plant growth [55, 74, 75, 83, 90, 92–96]. Similarly, we also observed positive correlations between the abundance of fertilization-enriched taxa and crop productivity in each cropping system (Supplementary Table S4 and Supplementary Fig. S9). Collectively, these results suggest that long-term fertilization stimulates various bacterial taxa in contrasting cropping systems, which ultimately provides positive feedback for crop productivity.

### Implications

For the establishment of green and highly efficient agriculture toward meeting the Sustainable Development Goals, it is important to understand how soil nutrients, microbiotas and crop productivity respond to long-term fertilization and their associated functional mechanisms in various cropping systems. Our results showed that long-term fertilization significantly and differentially affected soil bacterial community compositions in various cropping systems. Moreover, fertilization, particularly the NPM treatment, enlarged the differences in soil microbiome structure among cropping systems. Crop productivity was also closely associated with the abundance of fertilization-responsive taxa in the three cropping systems, highlighting the important role of such responsive taxa in supporting this highland agroecosystem.

The most striking results of this study include the reduced bacterial diversity and more fertilization-depleted taxa in the fertilization treatment for the GLR system, indicating that soil microbiomes in this crop rotation system are not very resistant to environmental fluctuations. According to the “insurance” theory, in which more diverse communities are more resistant and stable, thereby insuring ecosystems against declines in their functioning caused by environmental fluctuations [97]. Many previous studies also demonstrated that frequent crop-pea rotations could reduce soil microbial diversity and microbial activity, enrich pathogens, and decrease productivity [62–64, 98–100]. Taken together, our study demonstrates a strong need for balancing fertilization, nutrient pools, microbial diversity and productivity in legume-introduced diversified highland agroecosystem.

In this study, many of the statistical analyses are based on correlation analysis (e.g., redundancy analysis, network analysis, Mantel test, a priori structural equation model). Although these analyses provided the associations among the responses of bacterial communities to fertilization and soil properties, microbial activity, and crop productivity at the community level, they did not provide causality. We therefore recommend that explicitly manipulated experiments should be constructed to reveal the causal relationships in further studies.

## Supplementary Information

Supplementary Material

Supplementary Material

Supporting Information

## Data Availability

Sequencing data is available in the NCBI Sequence Read Archive repository under the accession number PRJNA853454. Any other relevant data are available from the corresponding author upon reasonable request.

## References

[1] Chen X, Cui Z, Fan M, Vitousek P, Zhao M, Ma W, et al. Producing more grain with lower environmental costs. Nature. 2014;514:486–9. 25186728 10.1038/nature13609

[2] Banerjee S, van der Heijden MGA. Soil microbiomes and one health. Nat Rev Microbiol. 2022;21:6–20. 35999468 10.1038/s41579-022-00779-w

[3] Singh JS, Pandey VC, Singh DP. Efficient soil microorganisms: a new dimension for sustainable agriculture and environmental development. Agric Ecosyst Environ. 2011;140:339–53.

[4] Li Z, Bai X, Jiao S, Li Y, Li P, Yang Y, et al. A simplified synthetic community rescues *Astragalus mongholicus* from root rot disease by activating plant-induced systemic resistance. Microbiome. 2021;9:217. 34732249 10.1186/s40168-021-01169-9 PMC8567675

[5] Wagg C, Schlaeppi K, Banerjee S, Kuramae EE, van der Heijden MGA. Fungal-bacterial diversity and microbiome complexity predict ecosystem functioning. Nat Commun. 2019;10:4841. 31649246 10.1038/s41467-019-12798-y PMC6813331

[6] Grigulis K, Lavorel S, Krainer U, Legay N, Baxendale C, Dumont M, et al. Relative contributions of plant traits and soil microbial properties to mountain grassland ecosystem services. J Ecol. 2013;101:47–57.

[7] Ji L, Ni K, Wu Z, Zhang J, Yi X, Yang X, et al. Effect of organic substitution rates on soil quality and fungal community composition in a tea plantation with long-term fertilization. Biol Fertil Soils. 2020;56:633–46.

[8] Liu J, Shu A, Song W, Shi W, Li M, Zhang W, et al. Long-term organic fertilizer substitution increases rice yield by improving soil properties and regulating soil bacteria. Geoderma. 2021;404:115287.

[9] Semenov MV, Krasnov GS, Semenov VM, van Bruggen AHC. Long-term fertilization rather than plant species shapes rhizosphere and bulk soil prokaryotic communities in agroecosystems. Appl Soil Ecol. 2020;154:103641.

[10] Song D, Dai X, Guo T, Cui J, Zhou W, Huang S, et al. Organic amendment regulates soil microbial biomass and activity in wheat-maize and wheat-soybean rotation systems. Agric Ecosyst Environ. 2022;333:107974.

[11] Li J, Yang Y, Wen J, Mo F, Liu Y. Continuous manure application strengthens the associations between soil microbial function and crop production: evidence from a 7-year multisite field experiment on the Guanzhong Plain. Agric Ecosyst Environ. 2022;338:108082.

[12] Liu H, Huang X, Tan W, Di H, Xu J, Li Y. High manure load reduces bacterial diversity and network complexity in a paddy soil under crop rotations. Soil Ecol Lett. 2020;2:104–19.

[13] Yang Y, Li X, Liu J, Zhou Z, Zhang T, Wang X. Bacterial diversity as affected by application of manure in red soils of subtropical China. Biol Fertil Soils. 2017;53:639–49.

[14] Obermeier MM, Minarsch EML, Durai Raj AC, Rineau F, Schröder P. Changes of soil-rhizosphere microbiota after organic amendment application in a *Hordeum vulgare L*. short-term greenhouse experiment. Plant Soil. 2020;455:489–506.

[15] Wang YF, Chen P, Wang FH, Han WX, Qiao M, Dong WX, et al. The ecological clusters of soil organisms drive the ecosystem multifunctionality under long-term fertilization. Environ Int. 2022;161:107133. 35149447 10.1016/j.envint.2022.107133

[16] Zhao M, Sun B, Wu L, Gao Q, Wang F, Wen C, et al. Zonal soil type determines soil microbial responses to Maize cropping and fertilization. mSystems. 2016;1:e00075–16. 27822546 10.1128/mSystems.00075-16 PMC5069962

[17] Jiang Y, Zhang J, Manuel DB, Op de Beeck M, Shahbaz M, et al. Rotation cropping and organic fertilizer jointly promote soil health and crop production. J Environ Manage. 2022;315:115190. 35526398 10.1016/j.jenvman.2022.115190

[18] Jiao S, Xu Y, Zhang J, Hao X, Lu Y. Core microbiota in agricultural soils and their potential associations with nutrient cycling. mSystems. 2019;4:e00313–18. 30944882 10.1128/mSystems.00313-18 PMC6435817

[19] Ramírez PB, Fuentes-Alburquenque S, Díez B, Vargas I, Bonilla CA. Soil microbial community responses to labile organic carbon fractions in relation to soil type and land use along a climate gradient. Soil Biol Biochem. 2020;141:107692.

[20] Vives-Peris V, de Ollas C, Gomez-Cadenas A, Perez-Clemente RM. Root exudates: from plant to rhizosphere and beyond. Plant Cell Rep. 2020;39:3–17. 31346716 10.1007/s00299-019-02447-5

[21] Zhang K, Maltais-Landry G, Liao HL. How soil biota regulate C cycling and soil C pools in diversified crop rotations. Soil Biol Biochem. 2021;156:108219.

[22] Hartmann M, Six J. Soil structure and microbiome functions in agroecosystems. Nat Rev Earth Environ. 2022;4:4–18.

[23] Ball CB, Bingham I, Rees RM, Watson CA, Litterick A. The role of crop rotations in determining soil structure and crop growth conditions. Can J Soil Sci. 2005;85:557–77.

[24] Esmaeilzadeh-Salestani K, Bahram M, Ghanbari Moheb Seraj R, Gohar D, Tohidfar M, Eremeev V, et al. Cropping systems with higher organic carbon promote soil microbial diversity. Agric Ecosyst Environ. 2021;319:107521.

[25] Ali A, Ghani MI, Elrys AS, Ding H, Iqbal M, Cheng Z, et al. Different cropping systems regulate the metabolic capabilities and potential ecological functions altered by soil microbiome structure in the plastic shed mono-cropped cucumber rhizosphere. Agric Ecosyst Environ. 2021;318:107486.

[26] Drinkwater LE, Wagoner P, Sarrantonio M. Legume-based cropping systems have reduced carbon and nitrogen losses. Nature. 1998;396:262–5.

[27] Coskun D, Britto DT, Shi W, Kronzucker HJ. How plant root exudates shape the nitrogen cycle. Trends Plant Sci. 2017;22:661–73. 28601419 10.1016/j.tplants.2017.05.004

[28] Wei X, Reich PB, Hobbie SE. Legumes regulate grassland soil N cycling and its response to variation in species diversity and N supply but not CO_2_. Glob Change Biol. 2019;25:2396–409. 10.1111/gcb.14636 30932274

[29] Chen Y, Du J, Li Y, Tang H, Yin Z, Yang L, et al. Evolutions and managements of soil microbial community structure drove by continuous cropping. Front Microbiol. 2022;13:839494. 35295291 10.3389/fmicb.2022.839494 PMC8920486

[30] Su F, Hao M, Wei X. Soil organic C and N dynamics as affected by 31 years cropping systems and fertilization in highland agroecosystems. Agric Ecosyst Environ. 2022;326:107769.

[31] Qiu L, Zhang Q, Zhu H, Reich PB, Banerjee S, van der Heijden MGA, et al. Erosion reduces soil microbial diversity, network complexity and multifunctionality. ISME J. 2021;15:2474–89. 33712698 10.1038/s41396-021-00913-1 PMC8319411

[32] Cui Y, Fang L, Guo X, Han F, Ju W, Ye L, et al. Natural grassland as the optimal pattern of vegetation restoration in arid and semi-arid regions: Evidence from nutrient limitation of soil microbes. Sci Total Environ. 2019;648:388–97. 30121038 10.1016/j.scitotenv.2018.08.173

[33] German DP, Weintraub MN, Grandy AS, Lauber CL, Rinkes ZL, Allison SD. Optimization of hydrolytic and oxidative enzyme methods for ecosystem studies. Soil Biol Biochem. 2011;43:1387–97.

[34] Wei X, Ma T, Wang Y, Wei Y, Hao M, Shao M, et al. Long-term fertilization increases the temperature sensitivity of OC mineralization in soil aggregates of a highland agroecosystem. Geoderma. 2016;272:1–9.

[35] Ochoa-Hueso R, Collins SL, Delgado-Baquerizo M, Hamonts K, Pockman WT, Sinsabaugh RL, et al. Drought consistently alters the composition of soil fungal and bacterial communities in grasslands from two continents. Glob Change Biol. 2018;24:2818–27. 10.1111/gcb.14113 29505170

[36] Ochoa-Hueso R, Arca V, Delgado-Baquerizo M, Hamonts K, Piñeiro J, Serrano-Grijalva L, et al. Links between soil microbial communities, functioning, and plant nutrition under altered rainfall in Australian grassland. Ecol Monogr. 2020;90:e01424.

[37] Callahan BJ, McMurdie PJ, Rosen MJ, Han AW, Johnson AJ, Holmes SP. DADA2: High-resolution sample inference from Illumina amplicon data. Nat Methods. 2016;13:581–3. 27214047 10.1038/nmeth.3869 PMC4927377

[38] Bolyen E, Rideout JR, Dillon MR, Bokulich NA, Abnet CC, Al-Ghalith GA, et al. Reproducible, interactive, scalable and extensible microbiome data science using QIIME 2. Nat Biotechnol. 2019;37:852–7. 31341288 10.1038/s41587-019-0209-9 PMC7015180

[39] Bates D, Mächler M, Bolker B, Walker S. Fitting linear mixed-effects models using lme4. J Stat Softw. 2015;67:1–48.

[40] Oksanen J, Blanchet FG, Friendly M, Kindt R, Legendre P, McGilinn D, et al. Vegan: community ecology package. R Package Version. 2018;2:4. https://CRAN.R-project.org/package=vegan.

[41] Love MI, Huber W, Anders S. Moderated estimation of fold change and dispersion for RNA-seq data with *DESeq2*. Genome Biol. 2014;15:550. 25516281 10.1186/s13059-014-0550-8 PMC4302049

[42] Benjamini Y, Hochberg Y. Controlling the false discovery rate: a practical and powerful approach to multiple testing. J R Statist Soc B. 1995;57:289–300.

[43] Grady KL, Sorensen JW, Stopnisek N, Guittar J, Shade A. Assembly and seasonality of core phyllosphere microbiota on perennial biofuel crops. Nat Commun. 2019;10:4135. 31515535 10.1038/s41467-019-11974-4 PMC6742659

[44] Friedman J, Alm EJ. Inferring correlation networks from genomic survey data. PLoS Comput Biol. 2012;8:e1002687. 23028285 10.1371/journal.pcbi.1002687 PMC3447976

[45] Csárdi G, Nepusz T. The igraph software package for complex network research. Inter Complex Syst. 2006;1695:1–9.

[46] Barberán A, Bates ST, Casamayor EO, Fierer N. Using network analysis to explore co-occurrence patterns in soil microbial communities. ISME J. 2012;6:343–51. 21900968 10.1038/ismej.2011.119 PMC3260507

[47] Berg G, Rybakova D, Fischer D, Cernava T, Champomier Vergès MC, et al. Microbiome definition re-visited: old concepts and new challenges. Microbiome. 2020;8:103. 32605663 10.1186/s40168-020-00875-0 PMC7329523

[48] Radujković D, van Diggelen R, Bobbink R, Weijters M, Harris J, Pawlett M, et al. Initial soil community drives heathland fungal community trajectory over multiple years through altered plant-soil interactions. New Phytol. 2020;225:2140–51. 31569277 10.1111/nph.16226

[49] Archer E. rfPermute: estimate permutation p-values for Random Forest importance metrics. R package version 1. 2016. https://CRAN.R-project.org/package=rfPermute.

[50] Augusto L, Boča A. Tree functional traits, forest biomass, and tree species diversity interact with site properties to drive forest soil carbon. Nat Commun. 2022;13:1097. 35233020 10.1038/s41467-022-28748-0 PMC8888738

[51] Saghaï A, Banjeree S, Degrune F, Edlinger A, García-Palacios P, Garland G, et al. Diversity of archaea and niche preferences among putative ammonia-oxidizing *Nitrososphaeria* dominating across European arable soils. Environ Microbiol. 2021;24:341–56. 34796612 10.1111/1462-2920.15830

[52] Mhlanga B, Ercoli L, Piazza G, Thierfelder C, Pellegrino E. Occurrence and diversity of arbuscular mycorrhizal fungi colonising off-season and in-season weeds and their relationship with maize yield under conservation agriculture. Biol Fertil Soils. 2022;58:917–35.

[53] Shipley B. The AIC model selection method applied to path analytic models compared using a d-separation test. Ecology. 2013;94:560–4. 23687881 10.1890/12-0976.1

[54] Lefcheck JS. piecewiseSEM: Piecewise structural equation modelling in r for ecology, evolution, and systematics. Methods Ecol Evol. 2016;7:573–9.

[55] Özbolat O, Sánchez-Navarro V, Zornoza R, Egea-Cortines M, Cuartero J, Ros M, et al. Long-term adoption of reduced tillage and green manure improves soil physicochemical properties and increases the abundance of beneficial bacteria in a Mediterranean rainfed almond orchard. Geoderma. 2023;429:116218.

[56] De Mastro F, Brunetti G, Traversa A, Blagodatskaya E. Fertilization promotes microbial growth and minimum tillage increases nutrient-acquiring enzyme activities in a semiarid agro-ecosystem. Appl Soil Ecol. 2022;177:104529.

[57] Song X, Fang C, Yuan ZQ, Li FM. Long-term growth of alfalfa increased soil organic matter accumulation and nutrient mineralization in a semi-arid environment. Front Environ Sci. 2021;9:649346.

[58] Gregorich E, Rochette P, Vandenbygaart A, Angers D. Greenhouse gas contributions of agricultural soils and potential mitigation practices in Eastern Canada. Soil Tillage Res. 2005;83:53–72.

[59] Li Y, Han C, Dong X, Sun S, Zhao C. Soil microbial communities of dryland legume plantations are more complex than non-legumes. Sci Total Environ. 2022;822:153560. 35114224 10.1016/j.scitotenv.2022.153560

[60] Sun R, Chen Y, Han W, Dong W, Zhang Y, Hu C, et al. Different contribution of species sorting and exogenous species immigration from manure to soil fungal diversity and community assemblage under long-term fertilization. Soil Biol Biochem. 2020;151:108049.

[61] Ye G, Banerjee S, He JZ, Fan J, Wang Z, Wei X, et al. Manure application increases microbiome complexity in soil aggregate fractions: results of an 18-year field experiment. Agric Ecosyst Environ. 2021;307:107249.

[62] Bainard LD, Navarro-Borrell A, Hamel C, Braun K, Hanson K, Gan Y. Increasing the frequency of pulses in crop rotations reduces soil fungal diversity and increases the proportion of fungal pathotrophs in a semiarid agroecosystem. Agric Ecosyst Environ. 2017;240:206–14.

[63] Niu Y, Bainard LD, May WE, Hossain Z, Hamel C, Gan Y. Intensified pulse rotations buildup pea rhizosphere pathogens in cereal and pulse based cropping systems. Front Microbiol. 2018;9:1909. 30190708 10.3389/fmicb.2018.01909 PMC6115495

[64] Woo SL, De Filippis F, Zotti M, Vandenberg A, Hucl P, Bonanomi G. Pea-wheat rotation affects Soil microbiota diversity, community structure, and soilborne pathogens. Microorganisms. 2022;10:370. 35208825 10.3390/microorganisms10020370 PMC8876268

[65] Lopez-Angulo J, de la Cruz M, Chacon-Labella J, Illuminati A, Matesanz S, Pescador DS, et al. The role of root community attributes in predicting soil fungal and bacterial community patterns. New Phytol. 2020;228:1070–82. 32557640 10.1111/nph.16754

[66] Na X, Yu H, Wang P, Zhu W, Niu Y, Huang J. Vegetation biomass and soil moisture coregulate bacterial community succession under altered precipitation regimes in a desert steppe in northwestern China. Soil Biol Biochem. 2019;136:107520.

[67] Tognetti PM, Prober SM, Baez S, Chaneton EJ, Firn J, Risch AC, et al. Negative effects of nitrogen override positive effects of phosphorus on grassland legumes worldwide. Proc Natl Acad Sci USA. 2021;118:e2023718118. 34260386 10.1073/pnas.2023718118 PMC8285913

[68] Banerjee S, Zhao C, Kirkby CA, Coggins S, Zhao S, Bissett A, et al. Microbial interkingdom associations across soil depths reveal network connectivity and keystone taxa linked to soil fine-fraction carbon content. Agric Ecosyst Environ. 2021;320:107559.

[69] Guo H, Dong P, Gao F, Huang L, Wang S, Wang R, et al. Sucrose addition directionally enhances bacterial community convergence and network stability of the shrimp culture system. NPJ Biofilms Microbiomes. 2022;8:22. 35410335 10.1038/s41522-022-00288-x PMC9001642

[70] Jiao S, Qi J, Jin C, Liu Y, Wang Y, Pan H, et al. Core phylotypes enhance the resistance of soil microbiome to environmental changes to maintain multifunctionality in agricultural ecosystems. Glob Change Biol. 2022;28:6653–64. 10.1111/gcb.16387 36002985

[71] Semenov MV, Krasnov GS, Semenov VM, Ksenofontova N, Zinyakova NB, van Bruggen AHC. Does fresh farmyard manure introduce surviving microbes into soil or activate soil-borne microbiota? J Environ Manage. 2021;294:113018. 34144322 10.1016/j.jenvman.2021.113018

[72] Zhang K, Maltais-Landry G, George S, Grabau ZJ, M.Small I, Wright D, et al. Long‑term sod‑based rotation promotes beneficial root microbiomes and increases crop productivity. Biol Fertil Soils. 2022;58:403–19.

[73] Pascual J, Huber KJ, Overmann J. *Pyrinomonadaceae* In: Whitman WB, editor. Bergey’s manual of systematics of archaea and bacteria. Hoboken: John Wiley & Sons; 2018. pp 1–4.

[74] Silva AMM, Estrada-Bonilla GA, Lopes CM, Matteoli FP, Cotta SR, Feiler HP, et al. Does organomineral fertilizer combined with phosphate-solubilizing bacteria in sugarcane modulate soil microbial community and functions? Microbial Ecol. 2021;84:539–55. 10.1007/s00248-021-01855-z 34498120

[75] Anderson CR, Condron LM, Clough TJ, Fiers M, Stewart A, Hill RA, et al. Biochar induced soil microbial community change: implications for biogeochemical cycling of carbon, nitrogen and phosphorus. Pedobiologia. 2011;54:309–20.

[76] Degelmann DM, Kolb S, Dumont M, Murrell JC, Drake HL. *Enterobacteriaceae* facilitate the anaerobic degradation of glucose by a forest soil. FEMS Microbiol Ecol. 2009;68:312–9. 19453494 10.1111/j.1574-6941.2009.00681.x

[77] Mehnaz S. Microbes - friends and foes of sugarcane. J Basic Microbiol. 2013;53:954–71. 23322584 10.1002/jobm.201200299

[78] Wüst PK, Horn MA, Drake HL. *Clostridiaceae* and *Enterobacteriaceae* as active fermenters in earthworm gut content. ISME J. 2011;5:92–106. 20613788 10.1038/ismej.2010.99 PMC3105676

[79] Kudjordjie EN, Hooshmand K, Sapkota R, Darban B, Fomsgaard IS, Nicolaisen M. *Fusarium oxysporum* disrupts microbiome-metabolome networks in *Arabidopsis thaliana* roots. Microbiol Spectr. 2022;10:e01226–22. 35766498 10.1128/spectrum.01226-22 PMC9430778

[80] Carrion VJ, Perez-Jaramillo J, Cordovez V, Tracanna V, de Hollander M, Ruiz-Buck D, et al. Pathogen-induced activation of disease-suppressive functions in the endophytic root microbiome. Science. 2019;366:606–12. 31672892 10.1126/science.aaw9285

[81] Deng X, Zhang N, Li Y, Zhu C, Qu B, Liu H, et al. Bio-organic soil amendment promotes the suppression of *Ralstonia solanacearum* by inducing changes in the functionality and composition of rhizosphere bacterial communities. New Phytol. 2022;235:1558–74. 35569105 10.1111/nph.18221

[82] Hosoda A, Kurosaki M, Kazama K, Murano H, Mizota C, Niizuma Y. Correlation between molecular microbial community and nitrogen cycling on ornithogenic soil affected by tsunami in Japan. Ecol Genet Genom. 2022;23:100114.

[83] Luo J, Tao Q, Jupa R, Liu Y, Wu K, Song Y, et al. Role of vertical transmission of shoot endophytes in root-associated microbiome assembly and heavy metal hyperaccumulation in *Sedum alfredii*. Environ Sci Technol. 2019;53:6954–63. 31145612 10.1021/acs.est.9b01093

[84] Thelusmond JR, Strathmann TJ, Cupples AM. The identification of carbamazepine biodegrading phylotypes and phylotypes sensitive to carbamazepine exposure in two soil microbial communities. Sci Total Environ. 2016;571:1241–52. 27481454 10.1016/j.scitotenv.2016.07.154

[85] Wu F, Jiao S, Hu J, Wu X, Wang B, Shen G, et al. Stronger impacts of long-term relative to short-term exposure to carbon nanomaterials on soil bacterial communities. J Hazard Mater. 2021;410:124550. 33223310 10.1016/j.jhazmat.2020.124550

[86] Aslani F, Tedersoo L, Põlme S, Knox O, Bahram M. Global patterns and determinants of bacterial communities associated with ectomycorrhizal root tips of Alnus species. Soil Biol Biochem. 2020;148:107923.

[87] Favet J, Lapanje A, Giongo A, Kennedy S, Aung YY, Cattaneo A, et al. Microbial hitchhikers on intercontinental dust: catching a lift in Chad. ISME J. 2013;7:850–67. 23254516 10.1038/ismej.2012.152 PMC3603401

[88] Madigan M, Cox SS, Stegeman RA. Nitrogen fixation and nitrogenase activities in members of the family *Rhodospirillaceae*. J Bacteriol. 1984;157:73–8. 6581158 10.1128/jb.157.1.73-78.1984 PMC215131

[89] Cai L, Gong X, Sun X, Li S, Yu X. Comparison of chemical and microbiological changes during the aerobic composting and vermicomposting of green waste. PLoS One. 2018;13:e0207494. 30475832 10.1371/journal.pone.0207494 PMC6261053

[90] Xiong C, Singh BK, He JZ, Han YL, Li PP, Wan LH, et al. Plant developmental stage drives the differentiation in ecological role of the maize microbiome. Microbiome. 2021;9:171. 34389047 10.1186/s40168-021-01118-6 PMC8364065

[91] Hahnke RL, Meier-Kolthoff JP, Garcia-Lopez M, Mukherjee S, Huntemann M, Ivanova NN, et al. Genome-based taxonomic classification of Bacteroidetes. Front Microbiol. 2016;7:2003. 28066339 10.3389/fmicb.2016.02003 PMC5167729

[92] Chen X, Wang J, You Y, Wang R, Chu S, Chi Y, et al. When nanoparticle and microbes meet: The effect of multi-walled carbon nanotubes on microbial community and nutrient cycling in hyperaccumulator system. J Hazard Mater. 2022;423:126947. 34481400 10.1016/j.jhazmat.2021.126947

[93] Diagne N, Arumugam K, Ngom M, Nambiar-Veetil M, Franche C, Narayanan KK, et al. Use of *Frankia* and actinorhizal plants for degraded lands reclamation. Biomed Res Int. 2013;2013:948258. 10.1155/2013/948258 PMC3844217 24350296

[94] Li Q, Xiang P, Zhang T, Wu Q, Bao Z, Tu W, et al. The effect of phosphate mining activities on rhizosphere bacterial communities of surrounding vegetables and crops. Sci Total Environ. 2022;821:153479. 35092784 10.1016/j.scitotenv.2022.153479

[95] Stone BW, Li J, Koch BJ, Blazewicz SJ, Dijkstra P, Hayer M, et al. Nutrients cause consolidation of soil carbon flux to small proportion of bacterial community. Nat Commun. 2021;12:3381. 34099669 10.1038/s41467-021-23676-x PMC8184982

[96] Urra J, Alkorta I, Lanzén A, Mijangos I, Garbisu C. The application of fresh and composted horse and chicken manure affects soil quality, microbial composition and antibiotic resistance. Appl Soil Ecol. 2019;135:73–84.

[97] Yachi S, Loreau M. Biodiversity and ecosystem productivity in a fluctuating environment: the insurance hypothesis. Proc Natl Acad Sci USA. 1999;96:1463–8. 9990046 10.1073/pnas.96.4.1463 PMC15485

[98] Nayyar A, Hamel C, Lafond G, Gossen BD, Hanson K, Germida J. Soil microbial quality associated with yield reduction in continuous-pea. Appl Soil Ecol. 2009;43:115–21.

[99] Nielsen DC, Vigil MF. Intensifying a semi-arid dryland crop rotation by replacing fallow with pea. Agric Water Manage. 2017;186:127–38.

[100] Yang T, Evans B, Bainard LD. Pulse frequency in crop rotations alters soil microbial community networks and the relative abundance of fungal plant pathogens. Front Microbiol. 2021;12:667394. 34122380 10.3389/fmicb.2021.667394 PMC8189174

