# Supplementary material for: Contrasting response of soil microbiomes to long-term fertilization in various highland cropping systems
Source: ISME Commun. 2023 Aug 18;3:81. doi: 10.1038/s43705-023-00286-w (PMC10439144; doi:10.1038/s43705-023-00286-w)
Supplement: Supplementary file 3 — Supporting Information [file 43705_2023_286_MOESM3_ESM.pdf]

Supporting Information

Table S1 PERMANOVA (adonis) analysis.

Table S2 Pairwise adonis and anosim analysis between different groups.

Table S3 Differential abundance analysis at ASV level

Table S4 Differential abundance analysis at family level

Fig. S1. Rarefaction curves showing the saturation of ASV saturation.

Fig. S2. A priori model showing the direct and indirect effects on microbial activity and crop productivity after long-term fertilization.

Fig. S3. Wheat yields in different fertilization treatments in WC and GLR systems.

Fig. S4. Venn diagram showing the numbers of shared/unique bacterial ASVs.

Fig. S5. Core taxa with occupancy of 100%.

Fig. S6. Topological properties of bacterial co-occurrence network.

Fig. S7. Topological properties of bacterial co-occurrence network including node, edge number and degree.

Fig. S8. Linking core taxa to microbial activity and crop productivity in different cropping systems.

Fig. S9. Spearman's correlations between fertilization-responsive ASVs.

Fig. S10. Linear regressions between network properties (node, edge and degrees) and soil properties (SOC, TN and NO_3^-) and bacterial α-diversity (Observed ASVs).

Fig. S11. Linear regressions between soil properties (SOC, NO_3^- and OP) and soil bacterial community composition.

Table S1 PERMANOVA (adonis) analysis showed the effects of fertilization under the same cropping system, or the effects of cropping system (CS) under the same fertilization treatment (FT).

	CS		FT		CS×FT	
	R^2	P	R^2	P	R^2	P
**All**	0.18	0.001	0.12	0.001	0.10	0.001
**Fertilization effects**						
AC	/	/	0.27	0.001	/	/
WC	/	/	0.24	0.001	/	/
GLR	/	/	0.27	0.001	/	/
**Cropping system effects**						
CK	0.28	0.001	/	/	/	/
P	0.30	0.001	/	/	/	/
NP	0.16	0.015	/	/	/	/
NPM	0.40	0.001	/	/	/	/

Note: CS, cropping system; FT, Fertilization treatment; AC, continuous alfalfa system; WC, continuous wheat system; GLR, grain-legume rotation system; CK, unfertilized control; P, phosphorus; NP, nitrogen + phosphorus; NPM, nitrogen + phosphorus + manure.

Table S2 Pairwise adonis and anosim analysis between different groups.

Group	Process	Adonis		Anosim	
		R^2	*P*	*R*	*P*
AC	CK vs. P	**0.17**	**0.002**	**0.32**	**0.007**
	CK vs. NPM	**0.26**	**0.002**	**0.74**	**0.002**
	P vs. NPM	**0.22**	**0.003**	**0.56**	**0.003**
WC	CK vs. P	0.08	0.653	-0.02	0.471
	CK vs. NP	0.13	0.107	0.18	0.082
	CK vs. NPM	**0.28**	**0.005**	**0.74**	**0.005**
	P vs. NP	0.11	0.143	0.10	0.143
	P vs. NPM	**0.25**	**0.005**	**0.67**	**0.004**
	NP vs. NPM	0.18	0.005	0.33	0.002
GLR	CK vs. P	0.12	0.089	0.09	0.174
	CK vs. NP	**0.23**	**0.005**	**0.54**	**0.008**
	CK vs. NPM	**0.29**	**0.005**	**0.78**	**0.002**
	P vs. NP	**0.16**	**0.033**	**0.34**	**0.030**
	P vs. NPM	**0.22**	**0.005**	**0.60**	**0.004**
	NP vs. NPM	**0.16**	**0.033**	**0.31**	**0.039**
CK	AC vs. WC	**0.29**	**0.003**	**0.86**	**0.003**
	AC vs. GLR	**0.27**	**0.003**	**0.63**	**0.002**
	WC vs. GLR	0.12	0.150	0.12	0.115
P	AC vs. WC	**0.32**	**0.002**	**0.93**	**0.005**
	AC vs. GLR	**0.28**	**0.002**	**0.57**	**0.002**
	WC vs. GLR	0.13	0.085	0.16	0.079
NP	WC vs. GLR	**0.16**	**0.019**	**0.40**	**0.009**
NPM	AC vs. WC	**0.38**	**0.002**	**0.99**	**0.003**
	AC vs. GLR	**0.38**	**0.002**	**0.88**	**0.003**
	WC vs. GLR	**0.25**	**0.002**	**0.60**	**0.004**

AC, continuous alfalfa system; WC, continuous wheat system; GLR, grain-legume rotation system; CK, unfertilized control; P, phosphorus; NP, nitrogen + phosphorus; NPM, nitrogen + phosphorus + manure

Table S3. Differential abundance analysis at ASV level between the unfertilized control and each fertilization treatment in AC, WC and GLR systems as determined by *DESeq2* package (FDR < 0.05)

Table S4. Differential abundance analysis at family level between the unfertilized control and each fertilization treatment in AC, WC and GLR systems as determined by *DESeq2* package (FDR < 0.05)

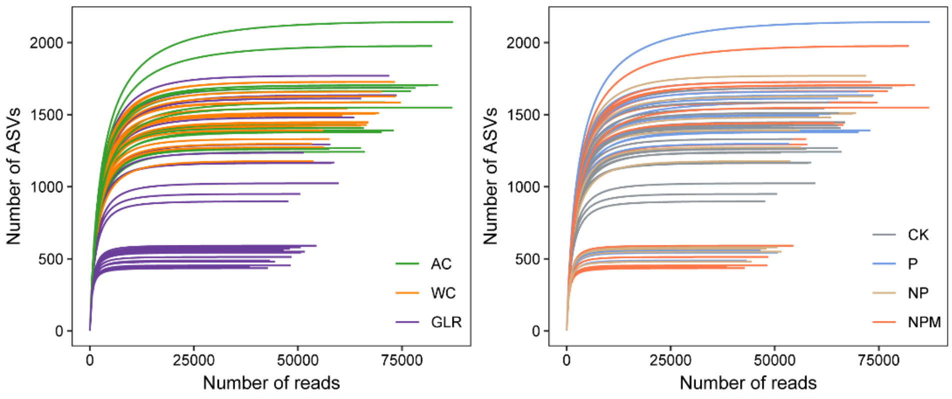

Fig. S1. Rarefaction curves based on the number of the observed ASVs, colored by cropping system **(a)** and fertilization **(b)**. Legend: AC, continuous alfalfa system; WC, continuous wheat system; GLR, grain-legume rotation system; CK, unfertilized control; P, phosphorus; NP, nitrogen + phosphorus; NPM, nitrogen + phosphorus + manure.

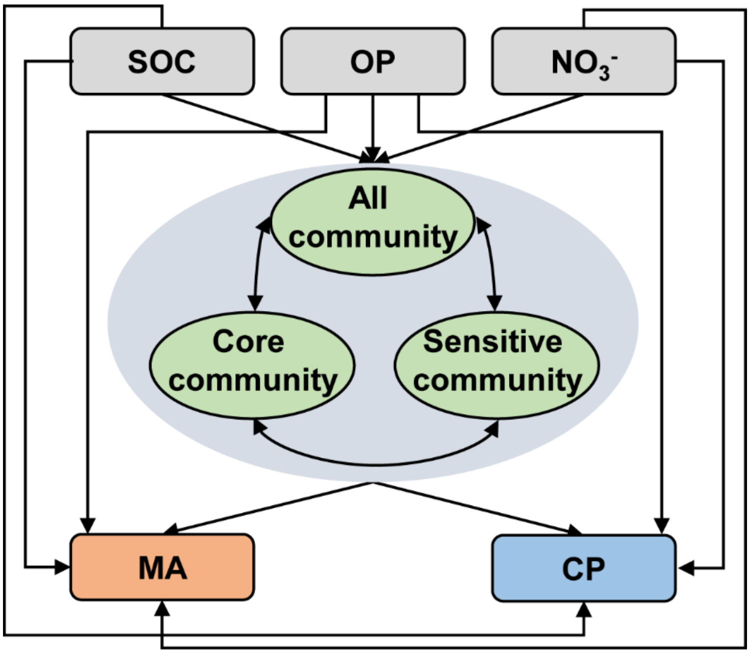

Fig. S2. A priori model showing the direct and indirect effects of the changes in soil properties (significant outputs from random forest) and composition of core or fertilization-sensitive community (first axis of principal coordinates analysis) on microbial activity and crop productivity after long-term fertilization. Legend: SOC, soil organic carbon; OP, available phosphorus; NO_3^-, nitrate; MA, microbial activity; CP, crop productivity. We hypothesized that changes in soil properties following long-term fertilization increased microbial activity and crop productivity by directly providing substrates for microbes and crops, and indirectly affecting bacterial community composition.

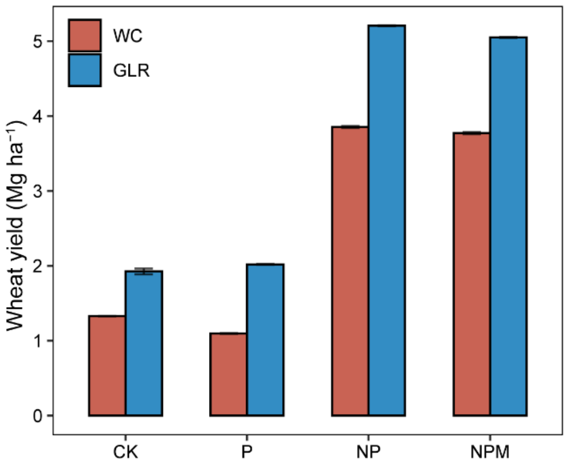

Fig. S3. Wheat yields in different fertilization treatments in WC and GLR systems. WC, continuous wheat system; GLR, grain-legume rotation system.

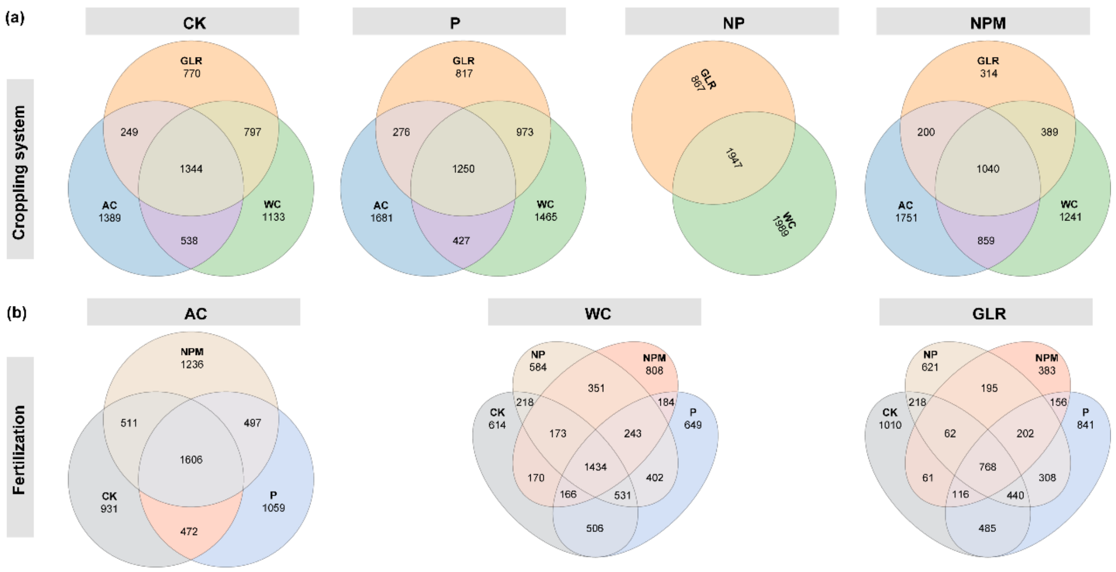

Fig. S4. Venn diagram showing the numbers of shared/unique bacterial ASVs among different cropping systems in each fertilization treatment **(a)** and among different fertilization treatments in each cropping system **(b)**. AC, continuous alfalfa system; WC, continuous wheat system; GLR, grain-legume rotation system; CK, unfertilized control; P, phosphorus; NP, nitrogen + phosphorus; NPM, nitrogen + phosphorus + manure.

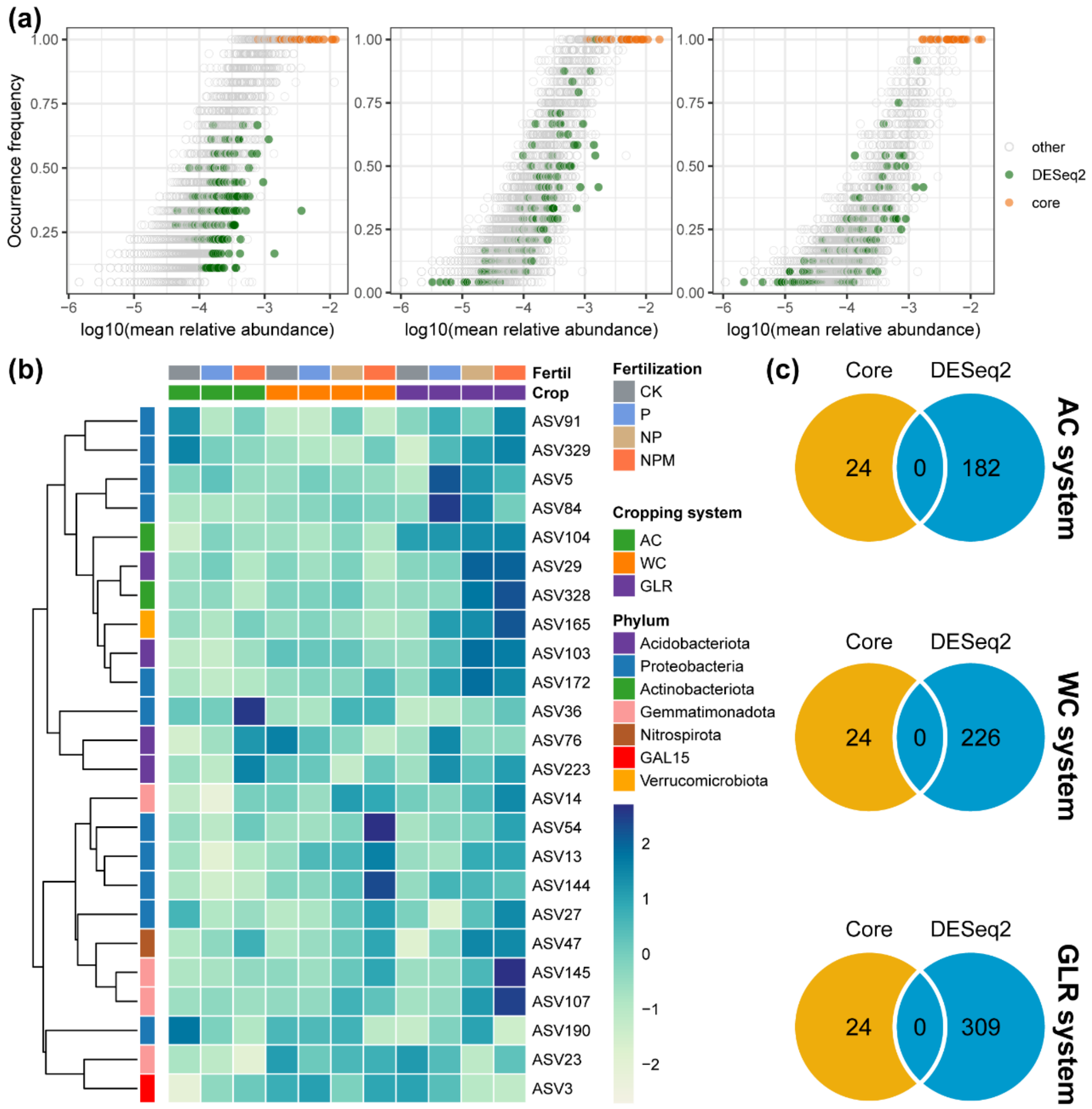

Fig. S5. Core taxa with occupancy of 100% were consistently detected in bacterial communities across all cropping systems and fertilization treatments. **(a)** Abundance-occupancy relationships of taxa. Taxa are colored for core, fertilization-sensitive, and other taxa. **(b)** Heatmaps showing the relative abundance of core ASVs. **(c)** Venn diagram showing the numbers of shared/unique bacterial ASVs among core taxa and fertilization-sensitive taxa (detached by *DESeq2* analysis) in AC, WC and GLR systems. Legend: AC, continuous alfalfa system; WC, continuous wheat system; GLR, grain-legume rotation system; CK, unfertilized control; P, phosphorus; NP, nitrogen + phosphorus; NPM, nitrogen + phosphorus + manure.

(a)

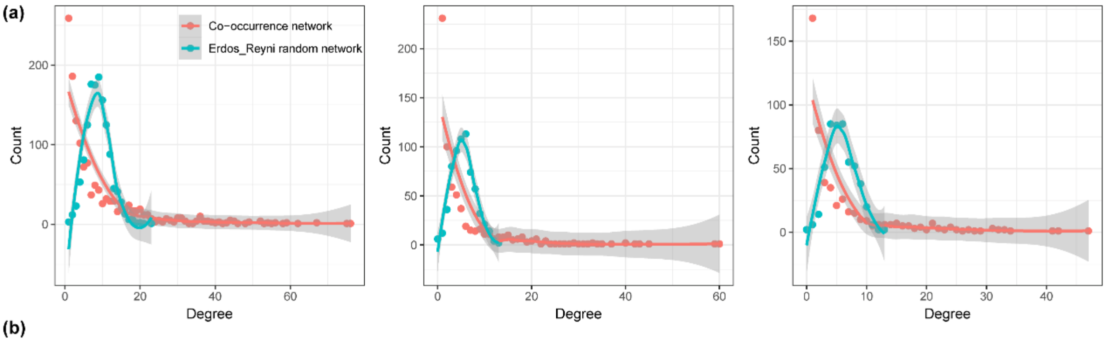

(b)

	Random network					Real network				
	avgPath	avgCC	Modularity	Diameter	Density	avgPath	avgCC	Modularity	Diameter	Density
**AC**	3.56 ± 0.002	0.007 ± 0.001	0.300±0.003	6.120 ± 0.325	0.007 ± 0.000	3.21	0.38	0.61	10.78	0.007
**WC**	3.97 ± 0.011	0.008 ± 0.002	0.409±0.005	7.783 ± 0.524	0.009 ± 0.000	3.49	0.37	0.65	10.39	0.008
**GLR**	3.73 ± 0.011	0.012 ± 0.002	0.395±0.006	7.212 ± 0.451	0.012 ± 0.000	3.28	0.43	0.65	8.94	0.012

Fig. S6. Topological properties of real and random bacterial co-occurrence network. **(a)** Power-law distribution pattern of the node degree. **(b)** Topological properties of bacterial real and random networks. avgPath, average path length; avgCC, average clustering coefficient; The random network of the same size was generated based on Erdös-Réyni model, and whose topological properties are calculated as the average value from 1000 Erdös-Réyni random networks.

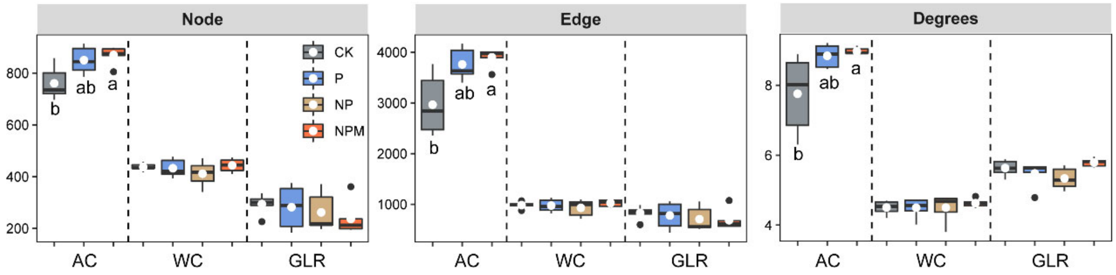

Fig. S7. Topological properties of bacterial co-occurrence network including node, edge number and degree as affected by cropping system and fertilization. Legend: AC, continuous alfalfa system; WC, continuous wheat system; GLR, grain-legume rotation system; P, phosphorus; NP, nitrogen + phosphorus; NPM, nitrogen + phosphorus + manure.

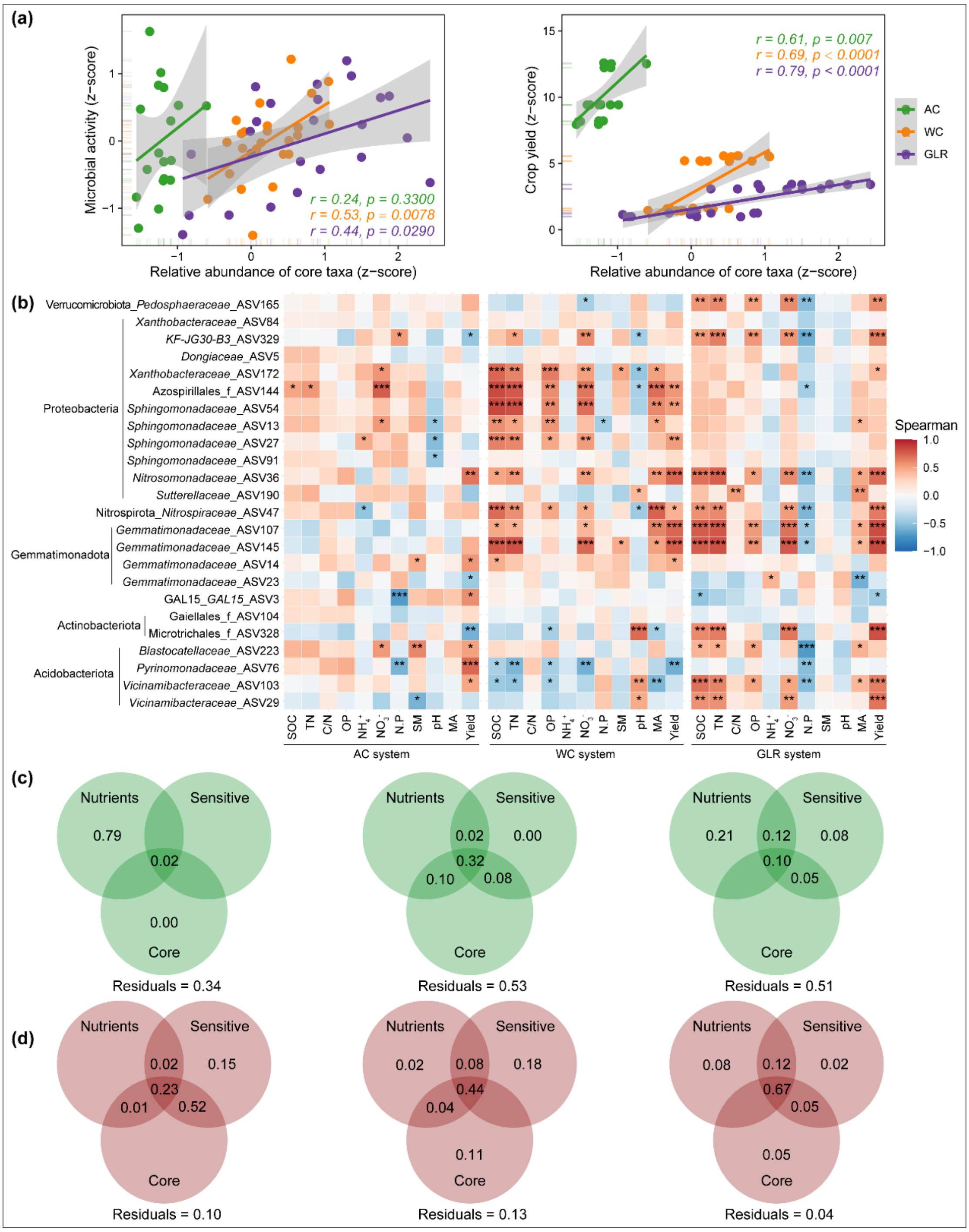

Fig. S8. Linking core taxa to microbial activity and crop productivity in different cropping systems. **(a)** The relationships between cumulative relative abundance of core taxa and microbial activity and crop productivity. **(b)** Spearman's correlations between each core taxa and soil properties, microbial activity and crop productivity. Variance partitioning analysis (VPA) showing the relative explanatory power of soil nutrients (SOC, NO_3^- and OP), core and sensitive community composition (first axis of principal coordinates analysis) in determining microbial activity **(c)** and crop productivity **(d)**. Legend: AC, continuous alfalfa system; WC, continuous wheat system; GLR, grain-legume rotation system.

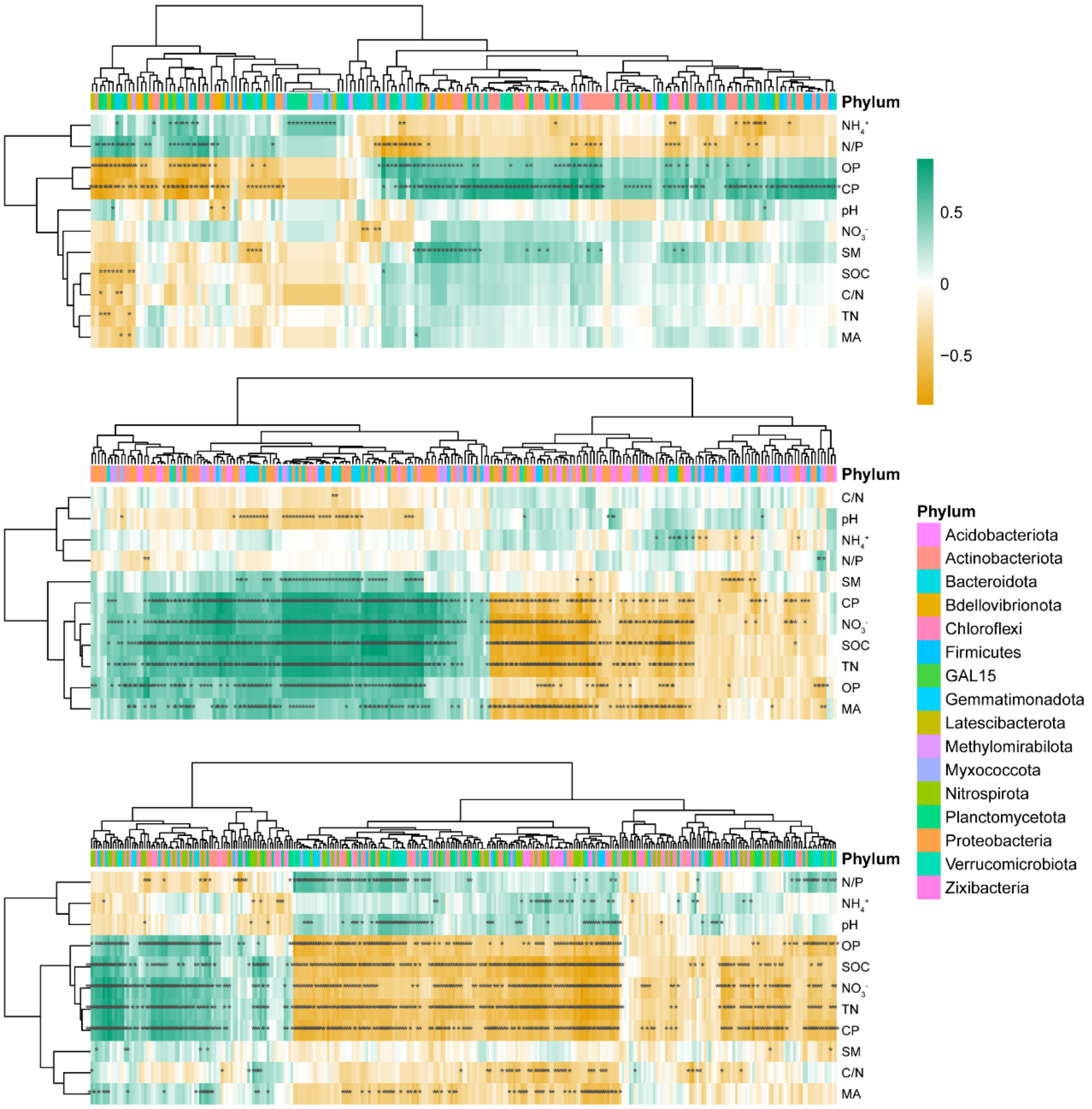

Fig. S9. Spearman's correlations between fertilization-responsive ASVs (detached by *DESeq2* analysis) and soil properties, microbial activities and crop productivity in AC **(a)**, WC **(b)** and GLR **(c)** systems. Legend: AC, continuous alfalfa system; WC, continuous wheat system; GLR, grain-legume rotation system; SOC, soil organic carbon; TN, total nitrogen; C/N, ratio of carbon to nitrogen; OP, available phosphorus; NH_4^+, ammonium; NO_3^-, nitrate; N/P, ratio of mineral nitrogen to available phosphorus; SM, soil moisture; pH, soil pH (H_2O); MA, microbial activity; CP, crop productivity.

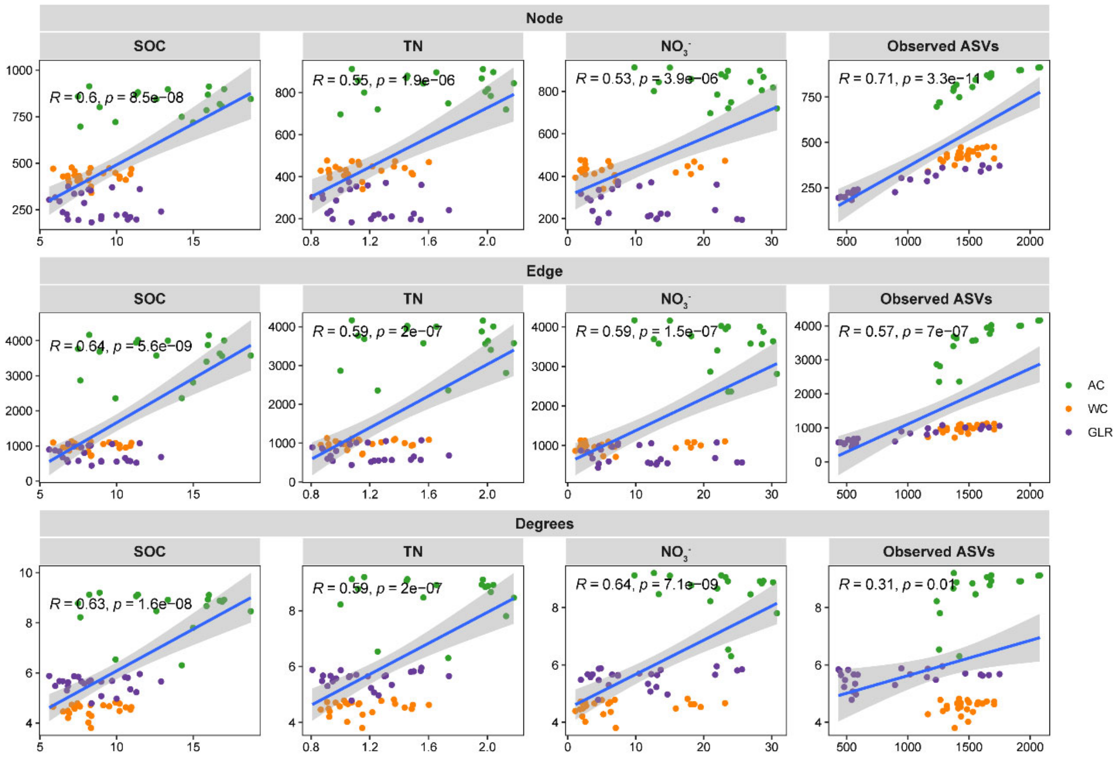

Fig. S10. Linear regressions between network properties (node, edge and degrees) and soil properties (SOC, TN and NO_3^-) and bacterial α-diversity (Observed ASVs). Legend: AC, continuous alfalfa system; WC, continuous wheat system; GLR, grain-legume rotation system; SOC, soil organic carbon; TN, total nitrogen; NO_3^-, nitrate.

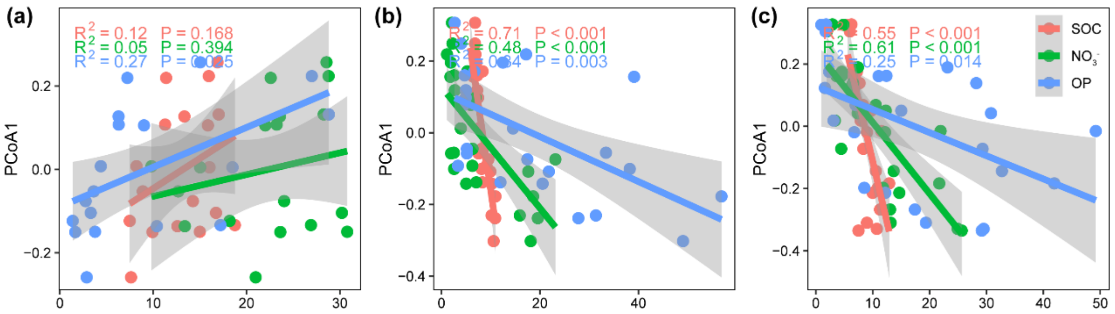

Fig. S11. Linear regressions between soil properties (SOC, NO_3^- and OP) and soil bacterial community composition (first axis of principal coordinates analysis) in AC **(a)**, WC **(b)** and GLR **(c)** systems. Legend: SOC, soil organic carbon; OP, available phosphorus; NO_3^-, nitrate.
